# Building Cell Selectivity into CPP-Mediated Strategies

**DOI:** 10.3390/ph3051456

**Published:** 2010-05-14

**Authors:** Irene Martín, Meritxell Teixidó, Ernest Giralt

**Affiliations:** 1 Institute for Research in Biomedicine (IRB Barcelona), Barcelona Science Park, Baldiri Reixac 10, Barcelona, Spain; E-Mail: irene.martin@irbbarcelona.org (I.M.); 2 Department of Organic Chemistry, University of Barcelona, Martí i Franquès 1-11, Barcelona, Spain

**Keywords:** cell-penetrating peptides (CPPs), targeted drug delivery, homing peptides, selective targeting, peptide transduction domains (PTDs)

## Abstract

There is a pressing need for more effective and selective therapies for cancer and other diseases. Consequently, much effort is being devoted to the development of alternative experimental approaches based on selective systems, which are designed to be specifically directed against target cells. In addition, a large number of highly potent therapeutic molecules are being discovered. However, they do not reach clinical trials because of their low delivery, poor specificity or their incapacity to bypass the plasma membrane. Cell-penetrating peptides (CPPs) are an open door for cell-impermeable compounds to reach intracellular targets. Putting all these together, research is sailing in the direction of the design of systems with the capacity to transport new drugs into a target cell. Some CPPs show cell type specificity while others require modifications or form part of more sophisticated drug delivery systems. In this review article we summarize several strategies for directed drug delivery involving CPPs that have been reported in the literature.

## 1. Introduction

**Figure 1:**
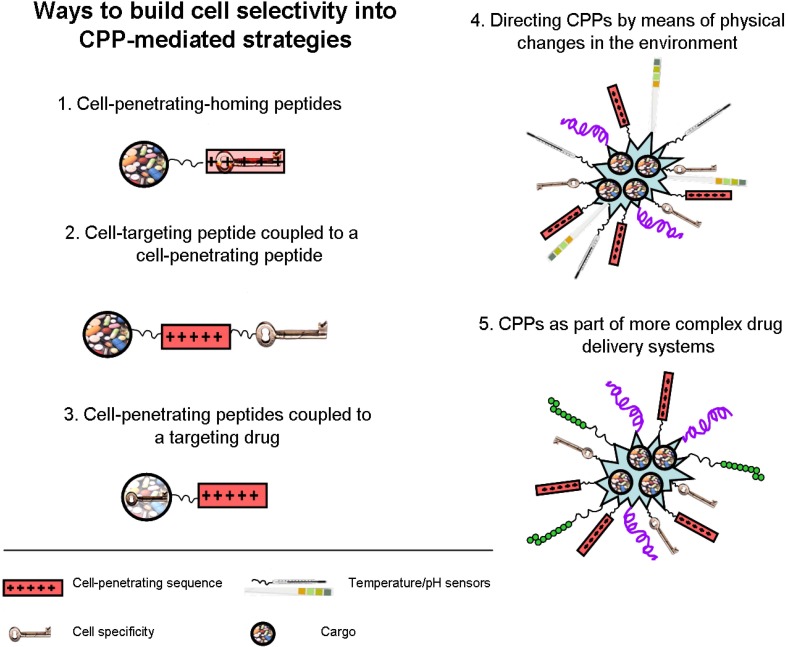
Strategies used to achieve cell selectivity.

Figure 1 summarizes different strategies for drug delivery involving CPPs. 

## 2. Cell-Penetrating-Homing Peptides

Cell-penetrating peptides (CPPs) [1,2,3,4,5,6,7,8] have been harnessed to translocate a wide range of molecules such as proteins, plasmids, peptide nucleic acids, short interfering RNA (siRNA), liposomes and nanoparticles across the cell membranes [9,10,11]. While CPPs provide a means through which cell-impermeable compounds can reach intracellular targets, conventional CPPs, like TAT or R_8_, can not deliver chemotherapeutic agents with cell specificity. Herein lays one of the major drawbacks of CPPs, their lack of cell specificity. This limitation hampers one of the most promising applications of CPPs; their use in the delivery of drug- and imaging agents would have a major relevance if they were able to target specific cells or tissues.

Each healthy or diseased tissue contains organ- or disease-specific molecular tags on its vasculature that constitute a vascular ‘zip code’ system [12]. The number of “homing peptides” that recognize specific types of cells is increasing every day. The advantage of these peptides is their capacity to recognize specific phenotypes and thus home onto a desired location. Furthermore, some of these peptides not only recognize the targeted cell but also have the capacity to translocate across its cellular membrane. These are the so called “cell-penetrating-homing peptides” (Table 1). The key feature of these peptides is that their sequences hold the target molecular address. Consequently, they offer great potential as vectors for drug delivery purposes, as they show the desired selectivity and the capacity to introduce cargos into a specific cell target. Cell-penetrating-homing peptides are usually small (no longer than 25–30 amino acids) non-immunogenic molecules and they show low cytotoxicity. Most of cell-penetrating homing peptides have been described using *in vivo* biopanning using phage display peptide libraries. The phage-display peptide library method was first developed by Parmley and Smith in 1988 [13]. This technique consists of the creation of a library of filamentous phages (such as M13) that express random peptides at the N-terminus of a protein (*i.e.* pIII) that is located at surface of the virus. The phage display peptide library is screened by biopanning. The following three general screening methods are currently used: (a) biopanning against purified cell surface membrane proteins [14]; (b) panning against intact cells [15]; and (c) *in vivo* selection by intravenous injection of phage-display libraries [12,16]. The bound phages are then eluted, amplified in *E. coli* and repanned a second time. The whole screening process is usually repeated a third time, in order to increase the affinity for the desired target. The amino acid sequence of the peptide displayed on each phagemid clone can be determined by DNA sequencing. This phage-display technology allows the detection of differentially expressed molecules and also differentially modified molecules. In addition, this technique does not require any previous knowledge of the molecular composition at the site of interest [12]. 

**Table 1:** Examples of some homing peptides and their cancer target [17].

Peptide	Homes to
AGR (CAGRRSAYC)	TRAMP (prostate)
LyP-2 (CNRRTKAGC)	K14-HPV16 (skin) tumor
	K14-HPV16/E2 (cervix) tumor
REA (CREAGRKAC)	TRAMP (prostate)
	PPC1 (prostate)
	M12 (prostate)
	DU145 (prostate)
	LNCaP (prostate)
	K14-HPV16/E2 (cervix)
	KRIB (osteosarcoma)
	MMTV-PyMT (breast)
LSD (CLSDGKRKC)	C8161 (melanoma)
	KRIB (osteosarcoma)

**Figure 2:**
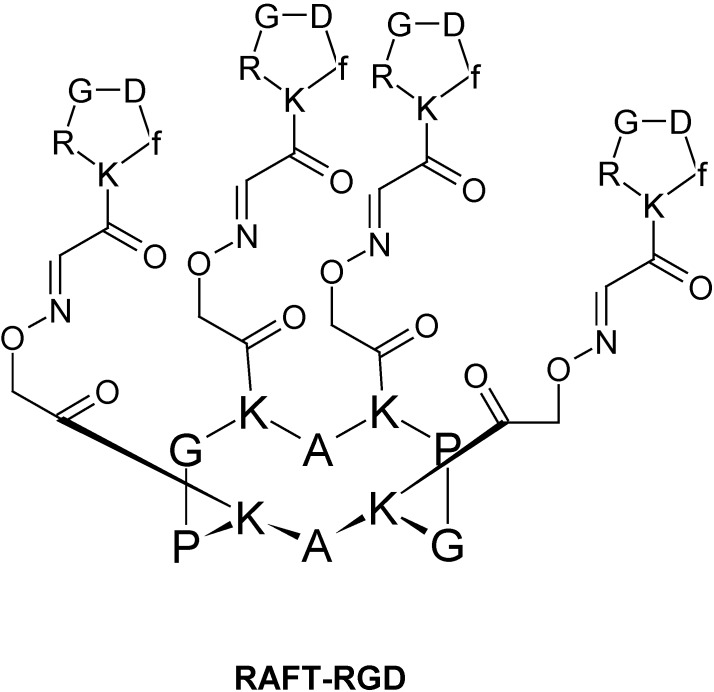
Chemical structure of RAFT-RGD [20].

One of the first was described to target tumor cells. It was in 1988 [18] when the internalization properties of RGD (Arg-Gly-Asp) peptides were first reported. These peptides recognize integrins, a family of cell-surface receptors that mediate the interaction of cells with the extracellular matrix, and are important for the migration and invasion of tumor cells. Integrin α_v_ß_3_ is overexpressed on neoendothelial cells and frequently on tumor cells [19]. Sancey *et al.* have recently developed a peptide-like constructs (RAFT-RGD) which targets integrin α_v_ß_3_ *in vitro* and *in vivo* [20]. It is based in a regioselectively addressable functionalized template (RAFT) cyclo-decapeptide scaffold developed by M. Mutter [21], able to present four cyclic RGD pentapeptide motifs (Figure 2). RAFT is a cyclic decapeptide (KKKPGKKKPG) with two orthogonally addressable domains pointing on either side of the cyclopeptide backbone. On the upper side, RAFT-RGD peptide has four copies of the cyclic RGDfK homing peptide while on the opposite face it has a fluorescent dye. The tetrameric RAFT-RGD was reported to have a ten-fold higher affinity for its soluble receptor integrin α_v_ß_3_ than the monomeric cRGD. Moreover, RAFT-RGD was rapidly internalized in small vesicles after 10 minutes. In fact, RAFT-RGD was proved to induce integrin α_v_ß_3 _ internalization. The observation that internalization is enhanced by RAFT-RGD allows it to be defined as a CPP. This research group revealed that the internalization of this peptide is through clathrin-mediated endocytosis. This finding contrasts with the trafficking route followed by the ß_1 _ integrin, which was shown to preferentially use a caveolae-dependent pathway. In summary, this selective peptide can be used for targeting α_v_ß_3_-integrin-expressing tumors and/or their microvasculature [20].

The neovasculature homing motif NGR was isolated from peptides upon screening of cyclic peptide libraries against the α5ß1 integrin [22]. This motif specifically binds to cells expressing aminopeptidase N (CD13), [23] a membrane-bound metallopeptidase that has multiple functions as a regulator of various hormones and cytokines, protein degradation, antigen presentation, cell proliferation, cell migration and angiogenesis. CD13 is barely expressed by the endothelium of normal blood vessels, and is up-regulated in angiogenic blood vessels [23]. Therefore, NGR-containing peptides can target endothelial cells and pericytes not only in tumors, but also in other pathological conditions, such as inflammation [24]. Various compounds and particles have been coupled or added synthetically to NGR peptides in order to increase their specificity towards neovasculature. The first example of an NGR peptide coupled to an anti-cancer drug was the conjugation of CNGRC peptide to doxorubicin [25]. This conjugate showed reduced toxicity and improved efficacy against human cancer xenografts in nude mice, compared with free doxorubicin. Liposomal formulations of doxorubicin can be targeted to tumors by coupling with linear peptides containing the CNGRC sequence [26,27]. Another example of the use of CNGRC as delivery vector is the complex formed by this cell-penetrating homing peptide and the mitochondrial disrupter peptide D(KLAKLAK)_2_. This peptide induces mitochondrial membrane disruption, rather than damaging eukaryotic plasma membranes [28,29,30]. This research group succeeded in demonstrating the apoptotic effect of the peptide construct in breast carcinoma xenografts in mice [28].

Special mention is given NGR-TNF, the result of the conjugation of this CNGRC peptide to the N-terminus of tumor necrosis factor α (TNF). This conjugate (named NRG-TNF) is currently being tested in Phase II clinical studies. Not only has this drug improved anti-tumor activity but the administration of only picograms of this complex (but not of TNF alone) exerts synergistic antitumor effects with various chemotherapeutic agents, such as doxorubicin, melphalan, cisplatin, paclitaxel and gemcitabine. The *modus operandi* of NGR-TNF is through the alteration of drug-penetrating barriers [31,32,33,34,35]. The biologic activity of other cytokines and anti-angiogenic molecules, such as IFNγ, IFNα2a, endostatin and tumstatin fragment has been improved by coupling to NGR peptides [36,37,38,39]. Targeted delivery of ultra-low doses (picogram range) of a recombinant IFNγ-CNGRC conjugate (IFNγ-NGR) to tumor vasculature overcomes major counter regulatory mechanisms and delays tumor growth in mice [39]. 

Bieker *et al.* [40] recently generated the fusion protein tTNF-NGR (the extracellular domain of tissue factor (truncated tissue factor, tTNF) and the peptide GNGRAHA, that contains the NGR motif), which targets tumor endothelial cells. tTNF-NGR inhibits tumor growth in mice by thrombotic occlusion of tumor vessels without any major side effects. It has also been reported to inhibit tumor perfusion in the first patients treated with this molecule [40]. With all these examples, NGR-containing peptides provide a good tool for tumor targeting of drugs and drug delivery systems.

F3 peptide is a 31 amino acid sequence derived from the nuclear protein high mobility group (HMG) protein 2 (HMGN2) [41]. HMGN2 is a highly conserved nucleosomal protein thought to be involved in unfolding higher-order chromatin structure and facilitating the transcriptional activation of mammalian genes [42]. The authors of the following work aimed to find markers shared by tumor vasculature and endothelial progenitor cells in the bone marrow. To do so they screened cDNA libraries displayed on phages, first on bone marrow cells *ex vivo* and then in tumor-bearing mice *in vivo*. F3 accumulates in the nuclei of tumor endothelial cells and tumor cells and has the capacity to carrying a cargo to the nuclei of the target cells. It recognizes a minor population of progenitor cell-like bone marrow cells, and it also binds to endothelial cells in tumors [41]. In addition, F3 appears to recognize a variety of tumor types; it binds not only the bone marrow subpopulation and tumor endothelial cells, but also systemically injected F3 recognizes a minor population in healthy skin and the gut. This peptide may be useful in targeting therapeutic agents into tumors. Furthermore, the capacity of F3 to carry a cargo into the cell nucleus makes it a promising tool for drug targeting applications, as multiple anti-cancer drugs exert their effect in the nucleus [41,43].

In Texas, Hong and Clayman described the isolation of a 12-mer peptide, HN-1 (TSPLNIHNGQKL) [44] able to translocate selectively across the cell membranes of human head and neck squamous cell carcinoma (HNSCC). The lack of specificity in current approaches to treat HNSCC produces dose-limiting toxicity, which has impeded the development of curative systemic approaches and also significant improvement in survival [45]. The authors of that study consider HN-1 as a potential shuttle of drugs into this kind of tumor. HN-1 is non-toxic, non-immunogenic in mice, and stable *in vivo*. Moreover, it protects its cargo during transit and accumulates efficiently within the tumor in 48 h [44]. This peptide may serve not only in cancer therapy, but also as a tumor diagnostic agent or imaging agent or it may even provide tumor-specificity to gene transfer approaches [44]. HN-1 is a novel peptide and the authors propose that its internalization is receptor-mediated. However, where is the limit between receptor-mediated endocytosis and specific adsorptive endocytosis of a CPP? Logically, all cell-penetrating homing peptides “recognize” some kind of molecule on the surface of the target cell. This recognition could lead to the classification of these peptides not as CPPs but as other kinds of peptides that are internalized into cells as a result of the interaction of a cellular receptor. We do not aim to discuss the tag of each peptide or their classification. We will consider homing cell-penetrating homing peptides as those peptides with the capacity to selectively recognize one or various types of cells and transport any kind of cargo across their cell membrane. 

Pep42 is a cyclic 13-mer oligopeptide (CTVALPGGYVRVC) that specifically binds to glucose-regulated protein 78 (GRP78) and internalizes into cancer cells. Given these properties, Pep42 is a promising vector for tumor cell-specific chemotherapy [46,47]. GRP78 was discovered in the late 1970s as a cellular protein induced by glucose starvation [48]. GRP78 expression is maintained at low basal levels in major adult organs such as the brain, lung, and heart, and is strongly induced in tumors [49]. In these cells, the overexpression of this intracellular chaperone and member of the heat shock protein 70 (HSP70) family provides a protective cellular response against stress conditions. Indeed, GRP78 is specifically overexpressed on the cancer cell surface. Normal GRP78 expression is maintained at low levels but is upregulated in a stress environment and induced in the tumor environment. Furthermore, in human cancer, higher expression of GRP78 has been related to greater pathological grade, recurrence and poor patient survival in breast, liver, prostate and colon cancers [49,50]. Pep42 is also internalized by receptor-mediated translocation (and clathrin-mediated endocytosis is its main mechanism of uptake); however, it has been referred to as a CPP [46,51]. Those authors demonstrated that Pep42 specifically recognizes GRP78 present on the cell surface of melanoma Me6654/2 cells, and after this recognition it is internalized. This targeted molecule is present only in cancer cells, as it has been previously shown that a number of human primary and cancer cell lines overexpress this glucose-regulated protein [49]. To assess the drug delivery properties of this new peptide, the authors conjugated Pep42 to a range of molecules with distinct biochemical properties and mechanisms of action. For instance, once again they chose the amphipathic apoptosis-inducing peptide D-(KLAKLAK)_2_. As seen above, this peptide exerts its action at the mitochondrial membrane. The other drug chosen was hematoporphyrin, a photosynthesizer that is active in the lysosomal compartment. These two drugs are imported to cells only in the presence of Pep42 [47]. A year later, Yoneda *et al*. evaluated Pep42-prodrug conjugates containing a cathepsin B-cleavable linker, in order to increase the release of drug inside the cancer cells, thereby facilitating endosomal release [51]. Their approach takes advantage of the presence of cathepsin B inside cell lysosomes. As previously described, Pep42 binds GRP78 receptor and is internalized with it. After the endosomal pathway, both molecules end up in the lysosomal compartment of cells. These researchers synthesized Pep42-drug conjugates containing cathepsin B cleavable linkers (Val-Cit motif in this case, which is highly stable in plasma and rapidly cleavable by cathepsin B [52]), and as drugs they chose the well known anticancer agents Taxol and doxorubicin. They did not perform *in vivo* assays, but the *in vitro* results in SJSA-1 (ostesarcoma cells, a GRP78-expressing cell line) are very promising. The delivery system induced higher cell death compared to treatment with the drug only [51]. 

This mitochondrial cytotoxic peptide, D-(KLAKLAK)_2_, was also coupled to the heptapeptide SMSIARL [53], isolated from an *in vivo* phage display peptide library, and specific target for the prostate vasculature. When this peptide was coupled to D-(KLAKLAK)_2_, only prostate tissue was destroyed. In prostate-cancer-prone transgenic TRAMP (transgenic adenocarcinoma of the mouse prostate model, [54]) this chimeric peptide postponed cancer development, thereby suggesting a potential alternative to the surgical approach used to treat this disease [53].

Azurin is a cupredoxin protein secreted by the microorganism *Pseudomonas aeruginosa*. Two peptides (amino acids 50 to 77 of azurin, p28 (LALSTAADMQGVVTDGMASGLDKDYLKPDD) and amino acids 50 to 67 of azurin, p18(LGLSTAADMQGVVTDGMASG)) have been described as a potential CPPs [55,56]. These peptides penetrate cancer cells without disrupting the cell membrane. Although still not fully understood, the uptake mechanism of p18 and p28 is energy-dependent and saturable. This mechanism involves caveolae and the Golgi complex, as nocodazole (which disrupts caveolae transport) and inhibitors of cholesterol mobilization inhibit the penetration of these peptides. The authors propose that the internalization of p18 and p28 follows more than one mechanism. But the main advantage of these peptides is that they preferentially enter cancer cells. The authors of that study showed an enhanced internalization rate in several tumoral cell lines *in vitro* compared with the corresponding non tumoral ones. In addition, these peptides inhibit cancer cell proliferation through cytostatic and cytotoxic mechanisms, as Azurin itself inhibits the growth of several human cancer lines *in vitro* [57,58,59]. Together, these peptides are not only capable of preferentially internalizing cancer cells, but can also exert a toxic effect on them and thereby prevent their proliferation.

Laakkonen together with Ruoslahti and coworkers have been intensively studying cell penetrating homing peptides [60,61]. They have performed *in vivo* biopanning using phage display peptide libraries to identify peptide ligands against vascular markers selectively expressed in tumor endothelium or lymph vessels. After several rounds of biopanning, some peptides have been selected. These are targeted to a specific site (homing to desired location) but some also have the capacity to internalize the targeted cells. In addition, some of these peptides are also able to destroy the targeted cell. LyP-1 is a nine-amino acid cyclic peptide (CGNKRTRGC) which was isolated in a screen using human MDA-MB-435 breast cancer xenografts [60]. Like F3, it recognizes a marker present in tumor cells and tumor endothelial cells. Furthermore, and unlike most vascular cell penetrating homing peptides, LyP-1 also recognizes tumor-associated lymphatic vessels. It recognizes the lymphatic vessel markers lymphatic vessel endothelial hyaluronic acid receptor-1 (LYVE-1, a transmembrane hyaluronic acid receptor) and podoplanin (a glomerular podocyte membrane protein), and also the vascular endothelial growth factor receptor 3 (VEGFR-3). LyP-1 accumulates in the nucleus of targeted cells, both in primary tumors and their metastatic lesions after intravenous injection, localizing preferentially in hypoxic areas. The homing of LyP-1 peptides is tumor type-specific; it accumulates in some tumors but not in others. In addition to MDA-MB-435 tumors, it homes to a transgenic prostate tumor (TRAMP) (Lakkonen, Bernasconi and Ruoslahti; unpublished observations), transgenic breast carcinoma (MMTV-PyMT) and, to lesser extent, KRIB osteosarcoma xenografts, but not to C8161 melanoma or HL-60 leukemia xenografts [61]. To further describe this extraordinary cell-penetrating homing peptide, it is mandatory to stress its capacity to trigger apoptosis of the cells to which it binds. Treatment of tumor cells with LyP-1 causes cell death. This effect is specific because cells that do not bind LyP-1 are not affected. The mechanism through which LyP-1 kills cells remains unknown, but the pro-apoptotic effect seems to be directed against tumor cells that are under stress, as LyP-1 colocalizes with a tissue hypoxia marker *in vivo*, and serum starvation enhances LyP-1 binding and internalization by cultured tumor cells *in vitro* [60]. Lymph vessels are highly relevant in tumor metastasis. Given that LyP-1 destroys the most deadly parts of tumors [12,17], this peptide could provide an extraordinary therapeutic strategy to target metastasis in the early stages of the process. 

Combining the positive selection of a peptide-bearing phage library on poorly differentiated colon carcinoma cells (HT29) with negative selection of the phage library on well-differentiated colon carcinoma cells (HCT116), Kimberly *et al*. [62] described a nine-amino acid, disulfide-constrained peptide, CPIEDRPMC (RPMrel). This peptide has been reported to target colon tumor tissues from four patients, without binding healthy colon tissue or other tissues such as lungs, lung sarcoma, liver, liver sarcoma or stomach. Furthermore, the authors report the efficacy of this novel peptide in transporting the mitochondrial toxin D(KLAKLAK)_2_ to these tumor colon cells, thereby inducing apoptosis and thus killing the cells [62]. Identification of a peptide that selectively enters colon cancer cells is very promising for developing new colon cancer diagnostic tools and therapeutic agents, such as RPMrel-D(KLAKLK)_2_. Colorectal cancer causes 655,000 deaths worldwide per year, and it is the third most common form of cancer and the third leading cause of cancer-related death in the western world. RPMrel could lead to the development of new direct targeting agents to fight against this disease.

Another example of a cell-penetrating homing peptide targets dorsal root ganglion (DRG) neurons [63]. Diseases affecting these neurons are rare, but are included in the differential diagnosis of peripheral sensory neuropathies [64]. In fact, neuropathic pain is a common symptom in various disorders. Although several pharmaceutical agents have been used to treat neuropathic pain, most are not specific and have limited efficacy. DRG neurons are classified as either large or small. J. Oi *et al*. isolated three peptides that recognize specific, defined sizes of DRG neurons [63]. Furthermore, these peptides have the capacity to internalize the targeted cells, and therefore the potential to become powerful tools for studying the subpopulations of DRG neurons and/or developing carriers of therapeutic agents against diseases involving specific subtypes of these neurons. The sequences of these peptides are the following: DRG1: SPGARAF; DRG2: DGPWRKM; and DRG3: FGQKASS. Interestingly, DRG1 and DRG3 internalize mostly in small neurons, while DGP2 shows preference towards larger ones. Experiments showed that DRG1 and DRG3 recognize distinct molecules in the surface of small DRG neurons. These results provide a powerful tool that will facilitate future research into the structural basis of neuronal cellular subpopulations and, more importantly, the generation of new molecular delivery systems that target DRG neurons *in vivo* [63]. 

Proline has unique properties among the 20 genetically encoded amino acids. Its pyrrolidine ring confers the molecule great rigidity, which leads to steric hindrance in peptidic backbones. Moreover, the conformation of the amide bonds cannot be stabilized by hydrogen bonds. Peptides containing a certain amount of proline in (polyprolines) adopt a well defined secondary structure, polyproline II (PPII), in pure water. PPII is a left-handed extended helix of 3.0 residues per turn (in contrast to the 3.3 residues which comprises the right-handed PPI formed by polyprolines in the presence of aliphatic alcohols). This architecture allows for the precise orientation of hydrophilic and hydrophobic moieties along different faces of the helix, and makes the structure soluble in aqueous media. Several proline-rich CPPs have been described to date [7,8,65]. However, there are two examples of cationic polyproline helices that have the capacity to internalize cells in a specific way: P11LR [66] and P11/LRR [67,68]. These peptides display a rigid type II polyproline helix backbone functionalized to contain six cationic moieties (believed to be essential in cell internalization) and two distinctive hydrophobic groups. Geisler *et al.* assayed the internalization of these two CPPs in seven cell lines (six cancerous and one non-cancerous) and described the selectivity of each for some of these cell lines [68]. For instance, P11LRR preferentially enters MCF-7 (breast cancer), and is slightly internalized also in KB 3-1 (HeLa derivative) and HT1080 (connective tissue cancer). However, it presented no internalization into the other cell types. P11F/LRR was most effectively internalized by KB 3-1 cells and to a lesser degree by MCF-7 cells compared to P11LRR. Several cytotoxicity assays were also performed. These indicated that P11LRR displays minimal cytotoxicity to the cell lines, while P11F/LRR is slightly more toxic [68]. Again, these CPPs show cell type preference for internalization. This finding was again exploited in the future design of directed drug delivery systems. In this case, both peptides were rationally designed [66] and not obtained from a direct *in vivo* observation, such as phage display biopanning. The selectivity shown by these proline-rich peptides should, in our opinion, be validated by *in vivo* experiments, to further develop their capacity to select the desired target in an *in vivo* context, which may differ greatly from *in vitro* observations.

As described above, phage display technology is a revolutionary tool to discover new highly specific peptides. This technique allows the selection of peptides with binding specificity to certain cells. In addition, *in vivo* phage display allows the binding of the peptides in a true physiological environment, in which diverse phenotype markers are expressed. Further screening of new phage libraries will undoubtedly lead to the discovery of highly selective peptides for certain diseases or even different stages of the same disease. Furthermore, some of these peptides would have the capacity to penetrate those cells, thereby providing the possibility to carry drugs or imaging agents, in order to directly target the affected area and thereby prevent the side effects caused by the non-specific drugs currently used. These cell-penetrating homing peptides are the minimal expression of a targeted drug delivery system [16,17,61,69].

## 3. Cell-Targeting Peptide Coupled to a CPP

Identifying a cell-specific peptide with cell-penetrating properties is not always easy. The capacity of a carrier to translocate across the cell membrane calls for many specific properties. The major drawback of the “classic” CPPs, such as TAT, *Penetratin*, and polyarginins, is their lack of specificity. However, they are powerful non-immunogenic tools to deliver cell-impermeable drugs to cells, such as cancer cells. Non-specific CPPs have been widely studied and their efficiency is improved almost yearly , and new CPPs are continually emerging. Many of these peptides are based on larger peptides or proteins present in nature, whose activity consists of breaking cellular membranes or penetrating cells [70,71]. Hence, their efficacy is high, an essential property for drug delivery systems. But having a strong internalization capacity commonly clashes with high specificity. Research in this field should make maximum use of all this knowledge. The following strategy for targeting CPPs is the design of a fusion peptide containing a cell-penetrating domain coupled to a targeting sequence, like for instance, a homing sequence.

**Figure 3:**
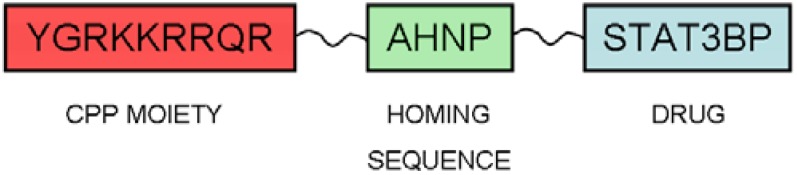
“Modular approach” design for targeted drug delivery systems [72].

With this in mind, Ming Tan and coworkers designed a novel CPP-based CPP specific for ErbB2-overexpressing breast cancer cells [72]. The ErbB2 gene is a member of the epidermal growth factor receptor family and is overexpressed in about 30% of breast cancer [73]. Current therapies for ErbB2-overexpressing cells are urgently required. This phenotype has been shown to increase the metastatic potential of human breast cancer and confers an increased resistance to some chemotherapeutic agents [74,75]. Once activated, ErbB2 activates STAT proteins (signal transducers and activators of transcription proteins). Once phosphorylated, STATs translocate into the nucleus, binding to STAT-specific DNA response elements, and thereby regulating gene expression [76]. It was found that ErbB2 activates STAT3, and this activation may contribute to ErbB2-induced transformation and tumor progression. Hence, specifically targeting STAT3 in ErbB2-overexpressing breast cancer cells that contain activated STAT3 may inhibit ErbB2-mediated malignant phenotypes. TAT peptide is one of the most studied CPPs and has been widely studied and conjugated to a variety of cargos to transport them into cells [77,78,79]. In this study, Ming Tan *et al.* conjugated an ErbB2 extracellular domain-binding peptide with a TAT-derived CPP in order to achieve target specificity of delivery [72]. They synthesized a less efficient version of TAT (YGRKKRRQR, called P3) as starting point for inducing cell specificity. This new version of TAT peptide was not as potent as the original TAT peptide; consequently, it did not internalize cells in such an efficient manner. This is an advantage for directing the system to the desired area, as it is not unspecifically internalized, and enters only the target cells. The addition of AHNP (a 12-amino acid anti HER-2/*neu* peptide mimetic which binds to ErbB2 with high affinity) at the C-terminus of this TAT-derived peptide rendered the translocation profile of the peptide ErbB2 selective both *in vitro* and *in vivo*. They also showed how this construct (P3-AHNP) efficiently and selectively delivered a STAT3-inhibiting peptide, named STAT3BP, and even selectively inhibited the growth of ErbB2-overexpressing cells *in vitro* [72,74]. Compared with ErbB2 low-expressing MDA-MB-435 xenografts, i.p. injected P3-AHNP-STAT3BP preferentially accumulated in 435.eB xenografts, which led to a greater reduction of proliferation and increased apoptosis and targeted inhibition of tumor growth [72]. This study confirms that the “modular approach” (Figure 3) in designing cell-specific CPPs is feasible. Replacing the “directing” moiety in each case (with any other molecular marker) may lead to a personalized therapy in which the “vehicle peptide” delivers the desired drug to the targeted cell.

PEGA (CPGPEGAGC) is a cyclic homing peptide that has previously been shown to accumulate in breast vasculature as well as in premalignant breast tissue and primary breast tumors [80]. It is thought that this peptide binds aminopeptidase P, a protein highly expressed in the membrane of breast tumor cells in mice [23]. PEGA is not a CPP. Langel’s group in Sweden, together with Myrberg from La Jolla, combined this tumor-specific homing peptide with the pVEC CPP. pVEC (LLIILRRRIRKQAHAHSK) is a well studied CPP derived from the murine sequence of the cell adhesion molecule vascular endothelial cadherin, amino acids 615–632. This CPP shows good internalization rates within several cell lines [81]. The combination of these two peptides showed internalization in tumor cells *in vivo* [82]. These researchers evaluated the specificity of the PEGA-pVEC construct by intravenously injecting the peptide labeled with fluorescein into MDA-MB-435 tumor-bearing mice. This peptide was detected in tumors but not in control tissue. Furthermore, conjugation of the peptide also with the nitrogen mustard (chlorambucil, Cbl, a cytotoxic agent that acts by alkylating and cross-linking DNA) produced higher efficacy of this drug for killing cells *in vitro*. Cbl is thought to be taken up by the cells by passive diffusion. A combination of this drug with the described chimera peptide resulted in an enhanced activity of this drug compared to its activity when administrated alone. This effect suggests that the new specific peptide construct could be useful in directed drug delivery to breast tumor tissue [82].

Two years later, in 2009, the chimeric peptide described above was improved. The system once again combined a breast tumor homing peptide with pVEC CPP. In this case, the homing peptide was CREKA, identified in breast tumors in MMTV-PyMT transgenic mice by using *in vivo* phage display [69]. This peptide recognizes clotted plasma proteins, homing selectively to tumor blood vessels and stroma. There, it binds to fibrin-like structures, but does not show internalizing properties. These researchers again combined the chimeric targeted carrier with the cytotoxic agent Cbl. This formulation showed enhanced internalization properties compared to the previous one (PEGA-pVEC), and was more convenient to synthesize. This system has improved characteristics and could also be a useful tool for directed drug delivery.

As these examples demonstrate, the combination of two or more peptides to achieve targeted delivery is already a reality. With these data in our hands, the range of possibilities is enormous. The conjugation of a homing peptide with a CPP to help in the translocation across the cellular membrane leads to a simple but efficient (and usually non-cytotoxic) drug delivery system. The number of homing peptides, as discussed above, increases yearly. In addition, the development of *in vivo* phage display technology could lead into a new “personalized” way of designing drug delivery systems.

For instance, Laakkonen and Ruoslahti have addressed new homing peptides that specifically recognize tumoral vasculature, tumoral cells or that are even able to distinguish between different stages of cancer [17]. For this purpose, they have worked extensively with phage display peptide libraries for *in vivo* screening, and have described several peptides of interest. Table 1 summarizes some examples within the lymphatic vasculature of several tumors. Combinations of targeting peptides and a CPP may provide a new approach to design specific drug delivery vectors. The level of specificity achieved would be selected by the type of tumor-homing peptide, which could be custom-design for each patient before the treatment. 

Several types of cancer overexpress certain surface molecules, including HER2 receptor in breast cancer, luteinizing hormone-releasing hormone receptor in most ovarian carcinomas, and CXC chemokine receptor 4 (CXCR4) receptor in multiple tumor types [83,84]. More than twenty types of cancer (for example breast cancer, ovarian cancer, glioma, pancreatic cancer, prostate cancer, cervical cancer, colon carcinoma, and renal cancer) overexpress CXCR4 [85,86]. In 2005, Steven F. Dowdy’s group [87] took advantage of these significant differences in the expression of tumoral cells versus healthy tissue to construct two chimeric peptides consisting of the CXCR4 receptor ligand, DV3 [88], bound to two proven transducible anticancer peptides, a p53-activating peptide (TATp53C'; [89]) and a cyclin-dependent kinase (cdk) 2 antagonist peptide (TAT-RxL; [90]). Thus, both anti-cancer peptides (named DV3-TATp53 and DV3-TAT-RxL) were specifically directed to the targeted cells (those overexpressing the CXCR4 receptor) and exerted their toxic effect without affecting non-tumoral cells [87]. 

By screening a random cyclic phage display library, Perea *et al.* identified a novel cyclic peptide, P15 (CWMSPRHLGTC), which abrogates CK2 phosphorylation by blocking the substrate [91]. This protease is considered a critical cancer target as it is frequently upregulated in many human tumors [92]. Direct inhibition of CK2 has been shown to induce apoptosis *in vitro* [93,94]. Therefore, CK2 phosphorylation constitutes a biochemical event that is a suitable target for the development of cancer therapeutics. P15 was fused to the TAT CPP to enhance the cellular uptake of the system. P15-Tat induced apoptosis, as evidenced by rapid caspase activation and cellular cytotoxicity in a variety of tumor cell lines. Furthermore, direct injection of P15-Tat into C57BL6 mice bearing day 7-established solid tumors resulted in substantial regression of the tumor mass [91]. 

Other examples of homing peptides can be found in the literature. For example, TLTYTWS peptide specifically binds to collagen IV modified by matrix metalloproteinase-2, (MMP-2) a essential protease involved in the process of angiogenesis [95]. This peptide is tumor-specific and inhibits angiogenesis in an *in vivo* assay in a concentration-dependent manner. It also significantly reduces endothelial differentiation *in vitro*. Peptides like this one, and all the other examples reported here, are potent candidates to specifically home to a wide range of tissues. Combining this homing capacity with that of CPPs may allow CPPs to be specifically directed to a desired location.

**Figure 4:**
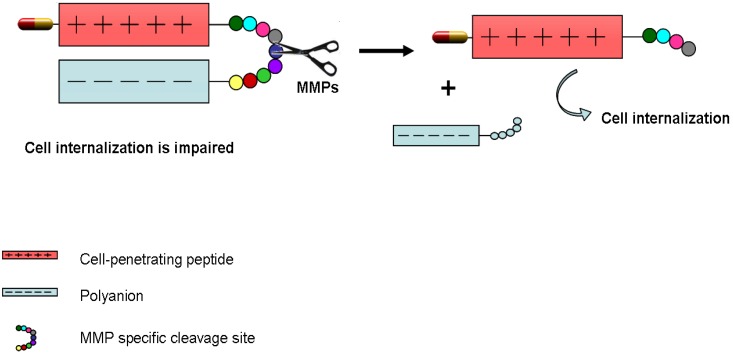
Schematic diagram of dormant CPP [96].

Tao Jiang *et al*. [96] took advantage of matrix metalloprotease (MMPs) oversecretion in cancer cells to create their targeted drug delivery system. MMPs are extracellular proteases that play a crucial role in extracellular matrix degradation, tissue invasion, and metastasis [97]. CPPs are known to enter cells as a result of their cationic nature, as they interact with the negatively charged cell surfaces [98]. The following system acts as a “dormant system” which is activated only upon reaching the desired tissue, in this case MMP-overexpressing cancer tissue. The positive charges of the CPP are initially masked by a polyanionic sequence. Upon reaching the tumoral tissue, overexpressed MMPs cleave the specific sequence that connects the CPP and the anionic sequence (Figure 4). Once the linker is cleaved, the acidic inhibitory domain drifts away, and the cationic CPP is free to carry its cargo into cells. This is a very interesting approach that benefits from an extracellular fate that occurs only in tumoral cells. The protease sequence could be designed in function of the kind of tumor or disease to be treated [96].

## 4. CPPs Coupled to a Targeting Drug

Until now we have addressed homing approaches based on peptide sequences, either inherent to the CPP sequence or provided by an adjacent homing peptide. This chapter will focus on another way of targeting a drug delivery system. In this case, the specificity is achieved by the drug, which has an effect only on the desired target. 

p53 is a protein known as the “guardian of the genome”, a transcription factor that is encoded by the *TP5 3* gene in humans. It regulates the cell cycle and functions as a tumor suppressor protein. p53 is the most frequently mutated gene in human cancer [99], and most of these mutations occur in the central DNA-binding region of the protein. This domain is regulated by its C-terminal sequence. Previous studies have reported that the addition of a chemically modified p53 C-terminal peptide (aa 363–393) restores *in vitro* sequence-specific DNA binding function to mutant p53-273 (Arg to His). Furthermore, intranuclear microinjection of this peptide into human colon cancer cells SW480 carrying an endogenous p53 mutant restores transcriptional activation of a p53-responsive reporter construct [100]. These results are very promising. But again, the bottleneck is the cellular uptake of this chemically modified p53 C-terminal peptide, which, due to its chemical structure, does not have the capacity to translocate across the cell membrane. Selivanova *et al*. designed a p53 C-terminal peptide (aa 361–382) fused to *Antennapedia* CPP to facilitate cellular uptake. They demonstrated inhibition of growth and induction of apoptosis in colon carcinoma cells with mutant p53 [101]. Fine’s team also reported that this construct (p53p-Ant) induced rapid apoptosis but this case in breast cancer cell lines carrying endogenous p53 mutations. The peptide was not toxic to non-malignant breast or colon cells. In a second study, this team showed that the same chimeric peptide induced apoptosis in human and rat glioma cells *in vitro* and *in vivo* [102,103,104]. They further studied the signaling mechanisms by which this protein acted in the cell. However, from the perspective concerning this review, the key feature of the system is the combination of two elements to target a desired cell and exert a pharmacological action. This strategy could be applied in the future treatment of cancers that have mutations in the DNA-binding domain of p53.

A similar approach was developed by Dowdy and coworkers for the treatment of HIV [105]. They designed what they have called a “Trojan horse” strategy to kill HIV-infected cells by exploiting HIV protease. In this case, the combination of TAT CPP with procaspase-3 (Casp3) protein leads to a new strategy to kill only HIV-infected cells, while leaving uninfected cells unharmed. The protein used was a modified procaspase 3 protein, TAT-Casp3, which transduces about 100% of infected and uninfected cells. However, Casp3 is modified and has the endogenous cleavage sites substituted by HIV proteolytic cleavage sites. Thus, TAT-Casp3 is activated only by HIV protease in infected cells, thereby resulting in apoptosis, whereas in uninfected cells it remains in the inactive zymogene form. These results are proof of concept for this kind of “intelligent” drug delivery system and could be applied to treat other diseases caused by pathogen proteases, by fine tuning the proteolytic cleavage sites of proteases involved in each disease, like hepatitis C virus, cytomegalovirus and malaria [105]. 

Most solid tumors grow in an environment that differs completely from that of healthy tissues. For instance, the pH surrounding tumoral tissue is lower and usually the tumoral mass has low pO_2._ As a result of the hypoxic conditions, the tumor is refractory to radiotherapy and anticancer chemotherapy. Tumoral cells that do not receive the proper amounts of oxygen try to adapt to their poor environment and undergo several changes. For example, under aerobic conditions, the expression of HIF-1 (hypoxia-inducible transcription factor 1) is barely detectable. Upon hypoxia, the expression of this protein is greatly increased, thereby inducing and activating glucose metabolism and glucose transport, and producing angiogenic and growth factors. HIF-1 is also involved in the prevention of apoptosis and activates genes involved in metastasis and tumor invasion [106]. Given all these data, HIF-1 could be considered a good target in cancer treatment. HIF-1 is a heterodimer formed by an α and a β subunit. These subunits have several domains. In particular, there is a unique oxygen-dependent degradation domain (ODD), which is responsible for the regulation of the oxygen-dependent degradation of the HIF-1α protein. This protein stabilizes HIF-1 in a hypoxic environment and degrades it immediately under normal oxygen conditions [107]. The objective of the following work was to design a drug able to destroy HIF-1-expressing cells. To do so, Masahiro Hiraoka’s group designed a PTD-ODD-Procaspase-3 (TOP3) fusion protein drug [107]. ODD stabilized the fusion protein only in hypoxic conditions, while at normal pO_2_ the complex was degraded. The ODD regulatory mechanism is dependent on the ubiquitin-proteasome system, which is located in the cell cytoplasm, so a PTD (protein transduction domain) was required to translocate the fusion protein into the cells. In this case, to translocate the system into the cell cytoplasm they used TAT peptide, which has been widely reported to transport proteins into cells [108]. Procaspase 3 (Casp3) is the functional part of the complex. When TOP3 is in HIF-1 expressing cells it is stable, and Casp3 is activated (initiator caspases are activated in hypoxic cells), thus leading to cell death. However, when the cell does not express enough HIF-1, the ODD domain leads to the ubiquitin degradation of the construct, thereby preventing the activation of Casp3 and thus triggering cell death. This final fusion protein product, TOP3, was systemically administered to mice bearing human pancreatic tumor xenografts. It suppressed tumor growth and reduced tumor size without any apparent side effects [107]. Given these observations, the specific imaging and targeting of HIF-1 active cells is feasible and may provide a novel approach to treat this kind of difficult cell in some tumors.

Hsp90 is an ATPase-directed molecular chaperone that oversees the control of protein folding quality during the cellular stress response, and whose client proteins are usually signaling molecules involved in cell proliferation and cell survival [109]. This chaperone is thought to play a key role in cancer, as it is commonly upregulated in tumors and may contribute to the correct folding of proteins synthesized under stress conditions, thereby helping the cell to adapt to unfavorable environments caused by tumor growth [110]. Furthermore, survivin is an essential regulator of cell proliferation, differentiation and apoptosis. This protein is overexpressed in cancer tissues, and it interacts with Hsp90 in order to maintain its correct conformation [111]. The following strategy to direct inhibit tumor growth and progression is based on the disruption of survivin-Hsp90 interaction, by means of a “target drug” that enters cells by a CPP [112]. Fortugno *et al*. identified a survivin sequence (K79-K90) that blocks the interaction between survivin and Hsp90 *in vitro* [111]. Working on this sequence, Janet Plescia and coworkers succeeded in minimizing the number of amino acids required to block this interaction, and created sepherdin (K79-L87), a molecule that interacts specifically with Hsp90, and also with the capacity to disrupt Hsp90-survivin complex. They also demonstrated that intracellular delivery of sepherdin induced tumor cell death. For this purpose, they fused the N terminal of the *Antennapedia* CPP, and showed 100% internalization in HeLa cells. They further demonstrated that this fusion protein was able not only to inhibit the interaction of Hsp90 and survivin (and thus inhibit the activity of this pro cancer protein) but also to bind the ATP pocket of Hsp90, thereby destabilizing all Hsp90 client proteins. This new peptidic drug had the capacity to disrupt two crucial cell survival pathways and thus has become a promising drug for cancer treatment. One of the most interesting parts of this work is the capacity of the fusion protein to induce apoptosis only in tumoral cells. This is the main objective of “targeted cancer therapy”, and was achieved by this group. Sepherdin did not reduce the viability of non-transformed cells; however, it effectively killed cancer cells [112]. This fusion protein was further tested *in vivo*, in human cancer models (prostate carcinoma PC3 cells grown as superficial tumors in immunocompromised mice). The administration of sepherdin when the tumor was palpable resulted in the almost complete ablation of tumor growth and did not affect other tissues such as lung, spleen or liver. In addition, sepherdin killed tumor cells far more rapidly and more potently than other Hsp90 antagonists currently used in clinical applications [112]. Consequently, sepherdin is considered a potential new specific drug for cancer treatment. 

Prion diseases are also referred to as transmissible spongiform encephalopathies (TSE). They occur in humans and animals and primarily affect the central nervous system. The hallmark of these diseases is the presence of microscopic vacuolization of the brain tissue, called spongiform degeneration (meaning that the tissue deteriorates and develops a spongy texture), and the presence of an abnormal protein, called scrapie prion protein (PrP^Sc^), prion or abnormal prion protein. Diseases involving PrP^Sc^, unlike other known infectious diseases, are believed to result from a change in the conformation or shape of a normal protein called cellular prion protein (PrP^C^), which is present in large amounts in the brain as well as in other tissues. The conformation of PrP^Sc^ makes it largely resistant to cellular degradation, and the accumulation in the brain is considered to cause neurodegeneration and death [113]. Interestingly, PrP^C ^ has several domains within its structure. It has some residues (1-22) that constitute a very hydrophobic signal sequence promoting entry into the endoplasmic reticulum (ER), and are usually cleaved before the protein reaches the cell surface, where is usually located in lipid raft regions. Residues 23–30 are positively charged, and form one of two independent nuclear localization signal-like segments of this protein. The basic segment of the prion protein has been described as a CPP [114] and confers solubility to the hydrophobic signal sequence. The prion protein-derived CPPs (PrP-CPPs) comprise the N-terminal signal peptide in mouse (residues 1–22) and residues 1–24 in bovine PrP, coupled to sequences 23–28 and 25–30, respectively (mPrP1 and bPrP1). These peptides transport hydrophobic cargos across cell membranes in a lipid raft-dependent macropinocytosis mechanism [115]. Lofgren and coworkers studied the capacity of various CPP to prevent PrP^C^ conversion to PrP^Sc^ [116]. This team found that peptides derived from the N-terminus of the unprocessed prion protein antagonizes prion infection. They worked *in vitro* using, GT1-1 cells, which are murine neuronal hypothalamic cells. These cells were infected with brain homogenate thus generating chronically prion-infected cell lines (Sc-GT1-1a and Sc-GT1-1b). mPrP_1–28 _ and bPrP_1–30_ strongly reduced PrP^Sc^ expression in both prion-infected cell lines, but did not affect PrP^C^ expression in non-infected GT1-1 cells [116]. Further research has been done to study the mechanisms by which these CPPs reduce PrP^Sc^ expression. However, the main conclusion of this work is that these CPPs show selective anti-prion activity, thus opening up an avenue for the development of new drugs against prion diseases.

We would like to add a final comment in this section of the review. We have described several strategies in which a CPP sequence helps a “target drug” to reach the intracellular compartment. One such approach involves taking advantage of specific proteases or overexpressed proteins. However, when considering “specific” drugs, mention of nucleic acid-based therapy is inevitable. Gene therapy is the maximum expression of a “target drug”, as it is sequence-specific. Various oligonucleotide derivatives have demonstrated remarkable potential for therapeutic application as gene regulators [117]. siRNA delivery is currently the focus of intensive research effort. It can specifically bind (after dicer processing) to mRNA sequences, thereby altering splicing sites and leading to modified proteins that may be useful in the treatment of diseases. However, nucleic acids are strongly negatively charged and are thus unable to cross the cell membrane. Descriptions of covalent and non-covalent strategies have been reported in which CPPs help siRNA to penetrate cells [10]. Many groups have demonstrated effective downregulation of gene expression by CPP-based siRNA delivery, although endosomal release continues to be a bottleneck and attempts to solve it are in course [10]. Some examples of CPP-siRNA as a potential delivery strategy are reviewed in the following studies [117,118,119,120,121,122,123,124,125]. The field of nucleic acid delivery is rapidly improving and undergoing further development. In fact, several chemical modifications have been performed to increase the stability of these molecules and reduce their size, thereby achieving improved binding specificity. For instance, peptide nucleic acids (PNAs) [123,126,127,128] are now widely used as phosphoramidate morpholino oligonucleotides (PMOs) are [129,130,131,132]. It is clear that complexing or conjugating oligonucleotides to CPPs is an efficient and non-toxic way to achieve intracellular delivery of pharmaceutical agents with significant biological activity. However, the main bottleneck of this delivery system is the endosomal escape. Further research effort should address this limiting step. 

## 5. Directing CPPs by Means of Physical Changes in the Environment

In this section we will focus on a different way of directing a drug delivery system. In this case, the system finds its specific location by a physical change in the target environment. Thus, to avoid unspecific uptake the CPP domain of the system is initially hidden until it reaches the target area. After reaching the desired tissue, this domain is “woken up” and fully activated to promote efficient internalization. Some examples of this kind of strategy are described below.

**Figure 5:**
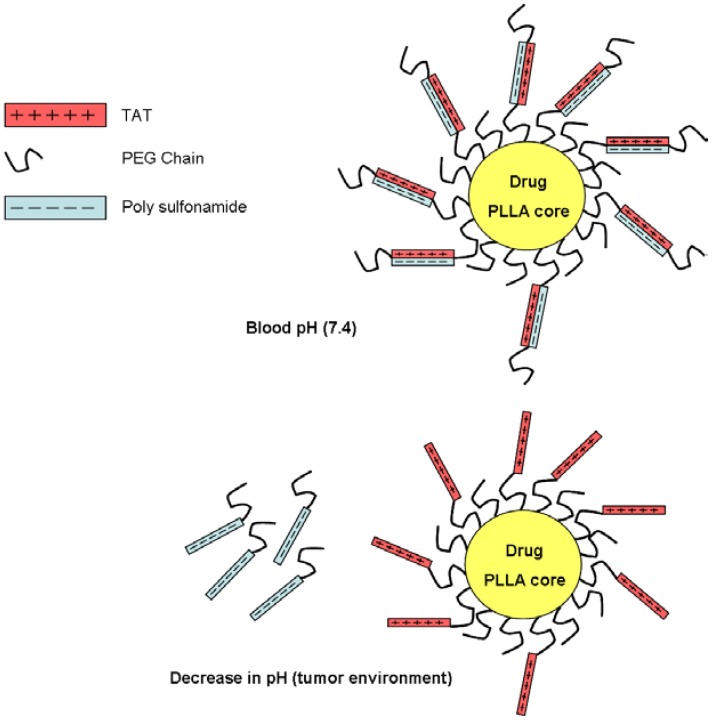
Schematic model for the drug delivery system proposed by Sethuraman and coworkers [133,134]. At pH 7.4 the sulfonamide is negatively charged, masking the positively charged TAT cell-penetrating peptide. But when the pH decreases in the vicinity of the tumor, the sulfonamide loses charge and detaches, thereby exposing TAT for interaction with the tumoral cells.

Sethuraman and coworkers designed a delivery system named “smart micellar nanoplatform” [133,134], which takes advantage of the low pH usually found in the tumor environment (pH below 7, as a result of lactic acid production in tumors [135,136,137]. This “smart micellar nanoplatform” has several components, which are shown in Figure 5. The core of the micelles is composed by poly(L-lactic acid) into which any chemotherapeutic agent can be incorporated. The core is decorated with polyethylene glycol (PEG) molecules that have TAT attached to them. TAT molecules electrostatically interact with polysulfonamide (PSD) molecules (negatively charged at pH = 7.4). Thus, TAT molecules are shielded and as a result the system cannot penetrate cells. When this system reaches the tumor site, the TAT micelles are exposed to the surroundings because PSD molecules become protonated and lose their negative charge. TAT then helps target the drug-loaded micelle to the tumoral cells, where the cytotoxic effect occurs. This system was tested *in vitro* using MCF-7 cells at a range of pH. Micelles loaded with fluorescent molecules penetrated cells only in an acidic environment, thereby proving the effectiveness of this intelligent system [134]. Nevertheless, further testing of this approach using cancer drugs and *in vivo* assays is required to evaluate its specificity and feasibility. 

**Figure 6:**
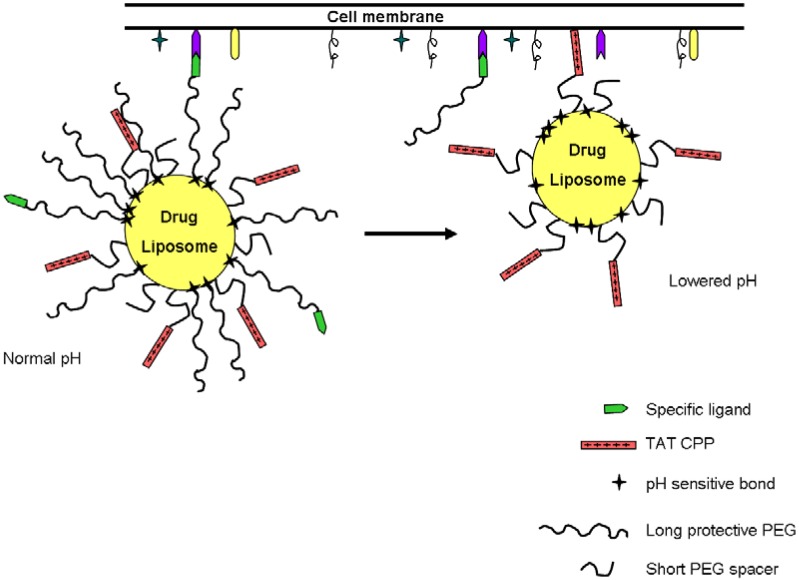
Interaction of the multifunctional pH-responsive pharmaceutical nanocarrier with the target cell [138]. A decrease of pH in the tumor environment induces the hydrolysis of the hydrazone bond, thereby exposing the CPP, which helps the delivery system to enter the tumoral cells.

In 2006, Torchilin’s group developed another strategy to target tumoral cells based on the decrease of pH in the tumoral tissue [138]. They worked with liposomes as pharmaceutical nanocarriers, whose surface holds several moieties (Figure 6). Until the complex reaches the low pH environment, TAT peptide is hidden by long-chain PEGs. Some of these chains also hold a ligand that helps to achieve the concentration of the complex at the target cells. Given that the long-chain PEGs are attached to the complex by means of pH labile bonds (hydrazone bonds), they are released from the complex in response to a lowering of the pH of the media. The TAT peptide is then exposed, and interacts with the cell surface, thereby contributing to the internalization of the liposome and the drug it holds [138]. This correct function of this system has been confirmed *in vitro* in NIH 3T3 fibroblasts at pH 8.0 and pH 5.0. One year later, the same team tested a similar drug delivery system *in vivo*. For this purpose, the pH-sensitive complex was administered directly into the tumor tissue by intratumoral injection [139]. This work represents a significant step towards the development of “intelligent” drug delivery systems, and is a striking example of a multifunctional drug delivery system.

One approach to treat primary tumors is by exposing them to high temperatures. Ablative therapies using temperatures above 43 °C of sufficient duration directly kill cells [140]. The application of mild hyperthermia (40–43 °C) is under clinical development as an adjunct with various established cancer treatments, such as radiotherapy and chemotherapy [130]. The following drug delivery system is temperature-sensitive and temperature is used to enhance the system selectivity to the target area. In conjunction with regional or local hyperthermia, temperature-sensitive delivery systems enhance their accumulation and penetration capacity in heated regions that contain the malignancy. To date, several temperature-sensitive peptides have been described [141,142,143,144]. They have been designed to change structures within a narrow thermal window around 40 °C. The transition between different secondary structures produces dramatic changes in peptide properties, and these changes should be reversible, as the peptide must return to its original state upon cooling to normal temperature. In 2005, Gene L. Bidwell and Drazen Raucher provided an example of a temperature-sensitive drug delivery system involving a CPP [145]. They designed an elastin-like polypeptide (Val-Pro-Gly-Xaa-Gly)_20_ coupled to *Penetratin* and to a peptide derived from helix 1 of the helix-loop-helix region of c-Myc (H1), known to inhibit c-Myc transcriptional function (Pen-ELP-H1). Elastin-like polymers are soluble in aqueous solution below their transition temperature (T_t_). However, when the temperature is raised above their T_t_, they undergo a phase transition and become insoluble, forming aggregates. In this case, heated MCF-7 cells showed notably greater uptake of the polypeptide compared to the same peptide in non-heat treated cells or compared to a non-responsive control peptide. This observation is attributed to the aggregation that takes place at high temperatures. Moreover, this aggregation effect is reversible. They also showed the cellular effect of peptide H1 [145,146]. The growth rate of HeLa and OVCAR-3 cells was slowed by this construct, thereby demonstrating the potential of this peptide in future treatment of tumors *in vivo*. The many favorable properties of the ELP sequence make it a good candidate drug delivery vector, as it has proved thermoresponsive properties, it is soluble at normal temperature and, in addition, it is expressed in bacteria. Large amounts of the ELP can be easily purified, and they can also be genetically modified in order to insert cell-penetrating sequences or therapeutic peptides or proteins. Applying high temperature to a tumoral mass could theoretically lead to an enhanced concentration of the delivery system in the region, thereby minimizing the side effects and improving cancer therapy.

In the following example the target tool of the system consists of a low-voltage electrical pulse [147]. The authors of that study proved that the internalization efficacy of TAT peptide coupled to glycogen synthase kinase-3 (GSK-3) was highly enhanced by the application of a low-voltage pulse (130–140V, depending on the cell line), without harming the cells. This study is preliminary; however, it demonstrates another way to target systems into cells [147]. Further studies could apply low-voltage pulses in regions containing malignant cells in order to increase drug delivery. 

We wish to make reference to a final strategy in which a drug delivery system takes advantage of a “physical” characteristic that is present only in the area to be directly targeted. In this case, the “physical” target is not an inherent property of the disease, but this delivery system is topically applied in the affected area (this system is used to treat inflammatory skin diseases). The drug consists of a polyarginine CPP conjugated to cyclosporine A (CsA) [148]. Skin diseases are usually treated with systemically administrated drugs, which may involve side effects. Topically administered drugs could be very useful for the treatment of skin diseases; however, the poor absorption as a result of the lack of skin permeability continues to be the main drawback of this approach. In this study, the use of CPPs enhanced drug absorption by the skin. Orally administered CsA is highly effective against a wide range of inflammatory skin diseases, such as psoriasis or atopic dermatitis [149,150]. CsA was conjugated to a heptamer of arginine through a linker designed to release the active drug at physiologic pH within tissue. This approach facilitated the penetration of CsA into mouse and human skin, the solubility of CsA was enhanced in water and effects of reduced inflammation were observed. These observations demonstrate the efficacy of CPPs to enhance skin absorption of agents, thus opening a new avenue in the field of skin disease treatment [148]. In fact, this treatment (PsorBan^®^) is currently in clinical trials and phase I trials were successful [122].

The literature provides other examples in which a CPP enhances drug absorption when applied locally. For instance, McCusker and coworkers described an original strategy for the treatment of allergic airway diseases [151]. They sought to inhibit STAT-6 protein, a key point target in the treatment of this disease as it is involved in the regulation of airway inflammation, mucus production and airway hyperresponsiveness [152]. However, targeting STAT-6 is complicated because of its intracellular location. This group designed a specific inhibitor peptide (STAT-6-IP) bound to TAT CPP. When delivered directly to the lungs of murine models, STAT-6-IP inhibited Ag-induced inflammatory responses, thereby inhibiting chemokine expression, mucus production and eosinophil influx into the lungs. Again, local application of a disease treatment consisting of a CPP coupled to a specific drug is a promising new therapeutic approach for the treatment of disease, in this case allergic rhinitis and asthma [151].

## 6. CPPs as Part of More Complex Drug Delivery Systems

The last section of this review addresses more complex drug delivery systems that target a specific location and that include a CPP, to increase the cellular uptake of the system. Some of these examples have been previously described, such as the a pH-sensitive drug delivery system system designed by V.P. Torchilin’s group [138].

In 2003, Heckl and coworkers published a new strategy to visualize prostate cancer using magnetic resonance imaging (MRI). Their objective was to develop a novel MRI contrast agent with the capacity to accumulate specifically in tumoral cells for relatively long periods [153]. Gadolinium (Gd^3+^) is a commonly used extracellular imaging agent for MRI, but it is not suitable for intracellular imaging. Assays using Gd^3+^ as an intracellular agent by means of microinjection into the cells [154], or conjugation with a CPP (TAT in this case [155]) had been reported. Apart from the lack of specificity, the main problem of these strategies is the rapid influx and efflux of the particles. To achieve a longer permanence of the Gd^3+^-complex in the cells, they constructed a Gd^3+^-complex bound to a PNA and connected it to *Antennapedia* CPP. The PNA was directed against the *c-myc* mRNA. This mRNA is highly expressed in tumoral cells [156]. They hypothesized that PNA coupling to *c-myc* mRNA would increase the life of the Gd^3+^, thereby providing this agent with specificity for MRI imaging. They verified the increased internalization of the complexes, on the basis of the presence of the CPP. Gd^3+ ^complex transport through the cell membrane using a CPP may provide an alternative approach for *in vivo* and *in vitro* application. They also observed that both *in vitro* (HeLa cells) and *in vivo* (prostate adenocarcinoma) the Gd^3+ ^ particles were retained longer than the Gd^3+^- Antennapedia particles without the *c-myc* PNA. The authors have been cautious in the interpretation of their results and have not directly related the presence of a complementary PNA for a highly expressed protein in the cells with the longer retention time of the particles inside the cells. They suggest that the longer retention of the complex may be due to a given property of the tumoral membrane cells. Thus, the slower efflux of the c-myc-specific Gd^3+ ^ complex would be independent of PNA design. Although further research in this field is required, these are promising results for specific intracellular imaging of tumors [153].

Liposomes have been widely studied for drug delivery in the treatment of many diseases, such as cancer and HIV [157,158,159]. In addition, cationic liposomes conjugated with DNA (lipoplexes) have been also investigated in preclinical and clinical trials because they have an *in vitro* transfection efficacy comparable to that of viral vectors [160]. The main drawback of these systems is, again, their lack of specificity. Despite having considerable potential, this approach still requires further development. Torchilin’s group has recently described a new strategy to target lipoplexes to ischemic cells [161]. The aim of the lipoplexes is to treat myocardial ischemia and myocardial infarction, two diseases in which gene therapy still holds a huge potential. There are enough data supporting that the overexpression of some genes in cardiac fibers protects them against ischemic injuries. For instance, overexpression of the fibroblast growth factor-2 in the heart increases resistance to ischemic injury in an isolated mouse heart model [162]. Shortly after this study, the gene transfer of HSP 70 was reported to protect cardiac functions under ischemia and reperfusion [163]. Improved myocardial perfusion and functional recovery have also been observed after gene transfer of VEGF, FGF and HGF [164,165]. Given all this information, directed gene delivery to cardiac cells would be a very good approach to treat this disease. Ko and coworkers have designed a new multifunctional drug delivery system as proof of concept for directed gene delivery into cardiac cells [161]. The system consists of cationic liposomes carrying pDNA encoding for green fluorescent protein (GFP) modified with TAT CPP (to increase cellular uptake) and the highly specific anti-myosin monoclonal antibody 2G4 (mAb 2G4). This antibody has demonstrated an excellent capacity to recognize and bind ischemic cells with damaged plasma membranes when intracellular myosin becomes exposed to the extracellular space [166]. Thus the lipoplexes concentrate in the desired area, and afterwards are internalized in the cells, thereby allowing gene transfer. Despite the solubility problems usually associated with intravenous injections of lipoplexes [167], the system described above has increased solubility because of the addition of some PEG3400-PE molecules to the system. This system was tested *in vitro* (cell culture with rat cardiomyocytes) and *in vivo* in an EMI model in rats and showed preferential accumulation of the construct in the ischemic areas of the infarcted rat heart [161]. These are promising results for the targeted delivery of genes; however, it would be interesting to see the effect of loading the therapeutic gene instead of GFP. Nevertheless, the field is undergoing certain improvement and in the near future these kinds of multicomponent systems are expected to be established as effective drug delivery systems in the treatment of many diseases.

The following strategy uses human serum albumin (HSA) as a natural carrier polymer. HSA has many advantages, such as long circulation times, high biocompatibility, high biodegradability and the possibility of being easily chemically modified [168,169]. In this case, the drug was SB202190, a p38MAP kinase inhibitor. Targeting p38MAP kinase is a current strategy widely studied for the treatment of chronic inflammatory diseases, as p38MAP kinases are responsible for activating endothelial cells in these diseases. These cells in turn secrete cytokines and chemokines that further promote leukocyte recruitment and infiltration [170]. The system also includes the cyclic RGDfK peptide, known to bind with high affinity and specificity to α_v_ß_3_ integrin, and previously defined as a CPP. This peptide confers selectivity and penetrating properties to the complex. α_v_ß_3_ integrin is overexpressed on angiogenic endothelium, which is part of the pathology of chronic inflammatory diseases such as rheumatoid arthritis and Crohn’s disease [171]. This non-toxic drug delivery system interfered with the inflammatory signaling cascade *in vitro*, thereby showing its potential for the treatment of inflammatory diseases. One year later, this same group published another HAS-based drug delivery system which also carried RGD CPP to target angiogenic endothelial and tumor cells and, in this case, Auristatin as an anti-cancer drug. *In vitro* results are encouraging [172], but in both cases further studies are required to evaluate the suitability of the systems for *in vivo* applications. 

There are several examples of drug delivery systems in which CPPs are used to increase the penetrability of the system into cells. In these cases, the specificity of the system is again through siRNA, which acts only in those cells expressing amounts of mRNA complementary to that siRNA. Some of these examples are reviewed in [173].

## 7. Future Directions

One of the major drawbacks in the field of drug delivery is the way of bypassing the cell membrane. Many potential therapeutic agents, such as nucleic acids, proteins and hydrophilic drugs, are not useful because of they are unable to enter cells, where they exert their therapeutic action. Several approaches have been developed to overcome this problem. For example, viral vectors are highly efficient for intracellular delivery [174,175], but they have disadvantages as they can induce a specific immune response and they are quite complicated structures. Further development is required to elucidate the biology underlying virus–host interactions. In addition, the *in vivo* transduction efficiency of viral vehicles requires improvement. The development of non-viral vehicles represents an easier, less expensive and safer alternative to viral vectors. Non-viral vehicles are simpler systems that can be fully controlled, and are mainly non-immunogenic. However, limitations such as low transfection efficiency and insufficient distribution to target cells *in vivo* have to be overcome. One of the most important features to be improved in the field of non-viral delivery systems is cell specificity. Many non-viral vectors are available for drug and gene delivery [118,176,177,178,179]. Recently different efforts in the direction of chemical modified viral vectors have proposed strategies such as chemo-virus and chimeric systems [180,181]. 

It is already more than 30 years since Frankel and Pabo described TAT in 1988 [182]. From then on, many cell-penetrating sequences have been reported, and many others have been improved. In addition, the capacity of these sequences to deliver a wide variety of cargos to cells, such as small peptides, proteins, DNA, siRNA, PNAs, and organic moieties, both *in vivo* and *in vitro* has been demonstrated. However, the main drawback of CPPs is that, despite their good cell-penetrating capacity and the fact that they are non-immunogenic and barely toxic, they do not show cell selectivity. The cellular uptake mechanism of CPPs is still not fully understood. There is a consensus that the first contacts between CPPs and the cell surface occurs through electrostatic interactions between the cationic peptide and the negatively charged cell surface [118]. Therefore the lack of specificity is a major issue to be solved before CPPs can be used for drug delivery. 

In this review, we have attempted to summarize examples of novel drug delivery systems. These systems share two main properties: they target a specific cell or tissue and their structure carries a CPP. 

Many of the strategies described have proved successful in *in vivo* experiments and therefore numerous clinical and preclinical studies of CPP-based delivery strategies are currently under evaluation. PsorBan^®^ is a cyclosporine-poly-arginine conjugate for the topical treatment of psoriasis [122]. Orally administrated cyclosporine A (CsA) is effective against a broad range of inflammatory skin diseases, including psoriasis. However systemic administration of this molecule has considerably side effects such as nephrotoxicity. Topical application of CsA would minimize side effects and would contribute to the treatment of these diseases. But topical application of CsA shows low effectiveness, due to a poor absorption. However, the conjugation of a CPP (heptaarginine) with CsA through a linker designed to release the active compound at tissue’s pH enhances its topical absorption, inhibiting cutaneous inflammation [148]. PsorBan^® ^ entered phase II trials in 2003 (CellGate, Inc.). Other CPP-based strategies for drug delivery are currently also undergoing clinical trials. For instance, KAI-9803 is being tested by Kai Pharmaceutical [183] as a TAT-protein kinase C inhibitor peptide modulator of protein kinase C for acute myocardial infarction and cerebral ischemia. In 2007, this peptide entered in phase II. Avi Biopharma is working on clinical development of CPPs for the *in vivo* steric block splicing correction using 6-aminohexanoic acid spaced oligoarginine [(R-Ahx-R)_4_]. It consists on a Morpholino oligo conjugated to the mentioned CPP [(RXR)_4_ XB CPP]. The goal of this construct is to prevent eventual blockage of a transplanted vein after cardiovascular bypass surgery [131,184]. Several companies (e.g. Traversa Inc., and Panomics Inc.) are currently evaluating CPPs in preclinical and clinical trials [118]. In addition, there are a great number of molecules conjugated to CPPs which are already in pre-clinical phases, getting optimized for future clinical trials [118,185]. In summary, CPPs are a reality for drug delivery. Inducing cell selectivity into CPP-mediated strategies will be the next step to improve current drug delivery systems, in order to decrease side effects and the amount of drug required to achieve a given intracellular target. The innovative range of strategies described in this review may soon enter clinical trials, which may contribute to furthering the field of drug delivery.

## References

[1] Hassane F.S., Saleh A., Abes R., Gait M., Lebleu B. (2009). Cell-penetrating-peptides: Overview and applications to the delivery of oligonucleotides. Cell. Mol. Life Sci..

[2] Andaloussi S.E., Guterstam P., Langel U. (2007). Assessing the delivery efficacy and internalization route of cell-penetrating peptides. Nat. Protocols.

[3] Pujals S., Fernandez-Carneado J., Kogan M.J., Martinez J., Cavelier F., Giralt E. (2006). Replacement of a proline with silaproline causes a 20-fold increase in the cellular uptake of a pro-rich peptide. J. Am. Chem. Soc..

[4] Futaki S., Nakase I., Tadokoro A., Takeuchi T., Jones A.T. (2007). Arginine-rich peptides and their internalization mechanisms. Biochem. Soc. Trans..

[5] Andaloussi S.E., Johansson H.J., Holm T., Langel U. (2007). A Novel cell-penetrating peptide, M918, for efficient delivery of proteins and Peptide Nucleic Acids. Mol. Ther..

[6] Gump J.M., Dowdy S.F. (2007). TAT transduction: The molecular mechanism and therapeutic prospects. Trends Mol. Med..

[7] Pujals S., Fernández-Carneado J., Ludevid M.D., Giralt E. (2008). D-SAP: A new, noncytotoxic, and fully protease resistant cell-penetrating peptide. Chem. Med. Chem..

[8] Pujals S., Giralt E. (2007). Proline-rich, amphipathic cell-penetrating peptides. Adv. Drug Delivery Rev..

[9] Torchilin V.P. (2007). Tatp-mediated intracellular delivery of pharmaceutical nanocarriers. Biochem. Soc. Trans..

[10] Endoh T., Ohtsuki T. (2009). Cellular siRNA delivery using cell-penetrating peptides modified for endosomal escape. Adv. Drug Delivery Rev..

[11] Järver P., Langel Ü. (2006). Cell-penetrating peptides-A brief introduction. Biochim. Biophys. Acta..

[12] Enbäck J., Laakkonen P. (2007). Tumour-homing peptides:Tools for targeting, imaging and destruction. Biochem. Soc. Trans..

[13] Parmley S.F., Smith G.P. (1988). Antibody-selectable filamentous fd phage vectors: Affinity purification of target genes. Gene.

[14] Fukuda M.N., Ohyama C., Lowitz K., Matsuo O., Pasqualini R., Ruoslahti E., Fukuda M. (2000). A peptide mimic of E-selectin ligand inhibits Sialyl Lewis X-dependent lung colonization of tumor cells. Cancer Res..

[15] Wang X., Cao B.B. (2009). Screening of specific internalization Fab fragment from Human Naive Phage Library by combinational bio-panning. Methods Mol. Biol..

[16] Kolonin M., Pasqualini R., Arap W. (2001). Molecular addresses in blood vessels as targets for therapy. Curr. Opin. Chem. Biol..

[17] Laakkonen P., Zhang L., Ruoslahti E. (2008). Peptide targeting of tumor lymph vessels. Ann. N. Y. Acad. Sci..

[18] Gehlsen K., Argraves W., Pierschbacher M., Ruoslahti E. (1988). Inhibition of *in vitro* tumor cell invasion by Arg-Gly-Asp-containing synthetic peptides. J. Cell Biol..

[19] Paolillo M., Russo M.A., Serra M., Colombo L., Schinelli S. (2009). Small molecule integrin antagonists in cancer therapy. Mini-Rev. Med. Chem..

[20] Lucie S., Elisabeth G., Stephanie F., Guy S., Amandine H., Corinne A.-R., Didier B., Catherine S., Alexei G., Pascal D., Jean-Luc C. (2009). Clustering and internalization of Integrin αvβ3 with a tetrameric RGD-synthetic peptide. Mol. Ther..

[21] Dumy P., Eggleston I.M., Cervigni S., Sila U., Sun X., Mutter M. (1995). A convenient synthesis of cyclic peptides as regioselectively addressable functionalized templates (RAFT). Tetrahedron Lett..

[22] Koivunen E., Gay D.A., Ruoslahti E. (1993). Selection of peptides binding to the alpha 5 beta 1 integrin from phage display library. J. Biol. Chem..

[23] Pasqualini R., Koivunen E., Kain R., Lahdenranta J., Sakamoto M., Stryhn A., Ashmun R. A., Shapiro L.H., Arap W., Ruoslahti E. (2000). Aminopeptidase N is a receptor for tumor-homing peptides and a target for inhibiting angiogenesis. Cancer Res..

[24] Corti A., Curnis F., Arap W., Pasqualini R. (2008). The neovasculature homing motif NGR: More than meets the eye. Blood.

[25] Arap W., Pasqualini R., Ruoslahti E. (1998). Cancer treatment by targeted drug delivery to tumor vasculature in a mouse model. Science.

[26] Pastorino F., Brignole C., Di Paolo D., Nico B., Pezzolo A., Marimpietri D., Pagnan G., Piccardi F., Cilli M., Longhi R., Ribatti D., Corti A., Allen T.M., Ponzoni M. (2006). Targeting liposomal chemotherapy via both tumor cell-specific and tumor vasculature-specific ligands potentiates therapeutic efficacy. Cancer Res..

[27] Pastorino F., Brignole C., Marimpietri D., Cilli M., Gambini C., Ribatti D., Longhi R., Allen T.M., Corti A., Ponzoni M. (2003). Vascular damage and anti-angiogenic effects of tumor vessel-targeted liposomal chemotherapy. Cancer Res..

[28] Ellerby H.M., Arap W., Ellerby L.M., Kain R., Andrusiak R., Rio G.D., Krajewski S., Lombardo C.R., Rao R., Ruoslahti E., Bredesen D.E., Pasqualini R. (1999). Anti-cancer activity of targeted pro-apoptotic peptides. Nat. Med..

[29] Law B., Quinti L., Choi Y., Weissleder R., Tung C.-H. (2006). A mitochondrial targeted fusion peptide exhibits remarkable cytotoxicity. Mol. Cancer Ther..

[30] Lemeshko V.V. (2009). Potential-dependent membrane permeabilization and mitochondrial aggregation caused by anticancer polyarginine-KLA peptides. Arch. Biochem. Biophys..

[31] Sacchi A., Gasparri A., Curnis F., Bellone M., Corti A. (2004). Crucial role for interferon γ in the synergism between tumor vasculature-targeted tumor necrosis factor-α (NGR-TNF) and doxorubicin. Cancer Res..

[32] Angelo C., Mirco P. (2004). Tumor vascular targeting with Tumor Necrosis Factor α and chemotherapeutic drugs. Ann. N. Y. Acad. Sci..

[33] Curnis F., Sacchi A., Corti A. (2002). Improving chemotherapeutic drug penetration in tumors by vascular targeting and barrier alteration. J. Clin. Invest..

[34] Zarovni N., Monaco L., Corti A. (2004). Inhibition of tumor growth by intramuscular injection of cDNA encoding Tumor Necrosis Factor α coupled to NGR and RGD tumor-homing peptides. Hum. Gene Ther..

[35] Sacchi A., Gasparri A., Gallo-Stampino C., Toma S., Curnis F., Corti A. (2006). Synergistic antitumor activity of Cisplatin, Paclitaxel, and Gemcitabine with tumor vasculature-targeted Tumor Necrosis Factor α. Clin. Cancer Res..

[36] Yumi Y., Sundaram R. (2005). Addition of an aminopeptidase N-binding sequence to human endostatin improves inhibition of ovarian carcinoma growth. Cancer.

[37] Meng J., Ma N., Yan Z., Han W., Zhang Y. (2006). NGR enhanced the anti-angiogenic activity of tum-5. J. Biochem..

[38] Meng J., Yan Z., Wu J., Li L., Xue X., Li M., Li W., Hao Q., Wan Y., Qin X., Zhang C., You Y., Han W., Zhang Y. (2007). High-yield expression, purification and characterization of tumor-targeted IFN-α2a. Cytotherapy.

[39] Curnis F., Gasparri A., Sacchi A., Cattaneo A., Magni F., Corti A. (2005). Targeted delivery of IFN-γ to tumor vessels uncouples antitumor from counterregulatory mechanisms. Cancer Res..

[40] Bieker R., Kessler T., Schwoppe C., Padro T., Persigehl T., Bremer C., Dreischaluck J., Kolkmeyer A., Heindel W., Mesters R.M., Berdel W.E. (2009). Infarction of tumor vessels by NGR-peptide-directed targeting of tissue factor: Experimental results and first-in-man experience. Blood.

[41] Porkka K., Laakkonen P., Hoffman J.A., Bernasconi M., Ruoslahti E. (2002). A fragment of the HMGN2 protein homes to the nuclei of tumor cells and tumor endothelial cells *in vivo*. Proc. Natl. Acad. Sci. USA.

[42] Bustin M. (1999). Regulation of DNA-dependent activities by the functional motifs of the high-mobility-group chromosomal proteins. Mol. Cell. Biol..

[43] Hurley L.H. (2002). DNA and its associated processes as targets for cancer therapy. Nat. Rev. Cancer..

[44] Hong F.D., Clayman G.L. (2000). Isolation of a peptide for targeted drug delivery into human head and neck solid tumors. Cancer Res..

[45] Hoekman K., van der Vijgh W.J.F., Vermorken J.B. (1999). Clinical and preclinical modulation of chemotherapy-induced toxicity in patients with cancer. Drugs.

[46] Kim Y., Lillo A.M., Steiniger S.C.J., Liu Y., Ballatore C., Anichini A., Mortarini R., Kaufmann G.F., Zhou B., Felding-Habermann B., Janda K.D. (2006). Targeting Heat Shock Proteins on cancer cells: Selection, characterization, and cell-penetrating properties of a peptidic GRP78 ligand. Biochemistry.

[47] Liu Y., Steiniger S.C.J., Kim Y., Kaufmann G.F., Felding-Habermann B., Janda K.D. (2007). Mechanistic studies of a peptidic GRP78 ligand for cancer cell-specific drug delivery. Mol. Pharmaceutics.

[48] Lee A.S. (2001). The glucose-regulated proteins: stress induction and clinical applications. Trends Biochem. Sci..

[49] Lee A.S. (2007). GRP78 induction in cancer: Therapeutic and prognostic implications. Cancer Res..

[50] Zhang J., Jiang Y., Jia Z., Li Q., Gong W., Wang L., Wei D., Yao J., Fang S., Xie K. (2006). Association of elevated GRP78 expression with increased lymph node metastasis and poor prognosis in patients with gastric cancer. Clin. Exp. Metastasis.

[51] Yoneda Y., Steiniger S.C.J., Čapková K., Mee J.M., Liu Y., Kaufmann G.F., Janda K.D. (2008). A cell-penetrating peptidic GRP78 ligand for tumor cell-specific prodrug therapy. Bioorg. Med. Chem. Lett..

[52] Dubowchik G.M., Firestone R.A. (1998). Cathepsin B-sensitive dipeptide prodrugs. 1. A model study of structural requirements for efficient release of doxorubicin. Bioorg. Med. Chem. Lett..

[53] Arap W., Haedicke W., Bernasconi M., Kain R., Rajotte D., Krajewski S., Ellerby H.M., Bredesen D.E., Pasqualini R., Ruoslahti E. (2002). Targeting the prostate for destruction through a vascular address. Proc. Natl. Acad. Sci. USA.

[54] Gingrich J.R., Barrios R.J., Morton R.A., Boyce B.F., DeMayo F.J., Finegold M.J., Angelopoulou R., Rosen J.M., Greenberg N.M. (1996). Metastatic prostate cancer in a transgenic mouse. Cancer Res..

[55] Tohru Y., Arsenio M.F., Vasu P., Laura B., Tapas K.D.G., Ananda M.C. (2005). Internalization of bacterial redox protein azurin in mammalian cells: Entry domain and specificity. Cell. Microbiol..

[56] Taylor B.N., Mehta R.R., Yamada T., Lekmine F., Christov K., Chakrabarty A.M., Green A., Bratescu L., Shilkaitis A., Beattie C.W., Das Gupta T.K. (2009). Noncationic peptides obtained from Azurin preferentially enter cancer cells. Cancer Res..

[57] Chaudhari A., Mahfouz M., Fialho A.M., Yamada T., Granja A.T., Zhu Y., Hashimoto W., Schlarb-Ridley B., Cho W., Gupta T.K.D., Chakrabarty A.M. (2007). Cupredoxin-cancer interrelationship: Azurin binding with EphB2, interference in EphB2 tyrosine phosphorylation, and inhibition of cancer growth. Biochemistry.

[58] Yang D.-S., Miao X.-D., Ye Z.-M., Feng J., Xu R.-Z., Huang X., Ge F.-F. (2005). Bacterial redox protein azurin induce apoptosis in human osteosarcoma U2OS cells. Pharmacol. Res..

[59] Yamada T., Hiraoka Y., Ikehata M., Kimbara K., Avner B.S., Das Gupta T.K., Chakrabarty A.M. (2004). Apoptosis or growth arrest: Modulation of tumor suppressor p53's specificity by bacterial redox protein azurin. Proc. Natl. Acad. Sci. USA.

[60] Laakkonen P., Åkerman M.E., Biliran H., Yang M., Ferrer F., Karpanen T., Hoffman R.M., Ruoslahti E. (2004). Antitumor activity of a homing peptide that targets tumor lymphatics and tumor cells. Proc. Natl. Acad. Sci. USA.

[61] Laakkonen P., Porkka K., Hoffman J.A., Ruoslahti E. (2002). A tumor-homing peptide with a targeting specificity related to lymphatic vessels. Nat. Med..

[62] Kimberly A.K., Jones D.A. (2003). Isolation of a colon tumor specific binding peptide using phage display selection. Neoplasia.

[63] Oi J., Terashima T., Kojima H., Fujimiya M., Maeda K., Arai R., Chan L., Yasuda H., Kashiwagi A., Kimura H. (2008). Isolation of specific peptides that home to dorsal root ganglion neurons in mice. Neurosci. Lett..

[64] Sghirlanzoni A., Pareyson D., Lauria G. (2005). Sensory neuron diseases. Lancet Neurol..

[65] Sadler K., Eom K.D., Yang J.-L., Dimitrova Y., Tam J.P. (2002). Translocating proline-rich peptides from the antimicrobial peptide Bactenecin 7. Biochemistry.

[66] Fillon Y.A., Anderson J.P., Chmielewski J. (2005). Cell-penetrating agents based on a polyproline helix scaffold. J. Am. Chem. Soc..

[67] Geisler I., Chmielewsk J. (2007). Probing length effects and mechanism of cell penetrating agents mounted on a polyproline helix scaffold. Bioorg. Med. Chem. Lett..

[68] Geisler I., Chmielewski J. (2009). Cationic amphiphilic polyproline helices: Side-chain variations and cell-specific internalization. Chem. Biol. Drug Des..

[69] Mäe M., Myrberg H., Andaloussi S.E., Langel Ü. (2009). Design of a tumor homing cell-penetrating peptide for drug delivery. Int. J. Pept. Res. Ther..

[70] Nakase I., Hirose H., Tanaka G., Tadokoro A., Kobayashi S., Takeuchi T., Futaki S. (2009). Cell-surface accumulation of flock house virus-derived peptide leads to efficient internalization via macropinocytosis. Mol. Ther..

[71] Andreu D., Merrifield R.B., Steiner H., Boman H.G. (2002). N-Terminal analogs of cecropin A: Synthesis, antibacterial activity, and conformational properties. Biochemistry.

[72] Tan M., Lan K.-H., Yao J., Lu C.-H., Sun M., Neal C.L., Lu J., Yu D. (2006). Selective inhibition of ErbB2-overexpressing breast cancer *in vivo* by a novel TAT-based ErbB2-targeting signal transducers and activators of Transcription 3-Blocking Peptide. Cancer Res..

[73] Yarden Y., Sliwkowski M.X. (2001). Untangling the ErbB signalling network. Nat. Rev. Mol. Cell Biol..

[74] Dihua Y., Mien-Chie H. (2000). Role of ErbB2 in breast cancer chemosensitivity. BioEssays.

[75] Tan M., Jing T., Lan K.-H., Neal C.L., Li P., Lee S., Fang D., Nagata Y., Liu J., Arlinghaus R., Hung M.-C., Yu D. (2002). Phosphorylation on Tyrosine-15 of p34Cdc2 by ErbB2 Inhibits p34Cdc2 Activation and is Involved in Resistance to Taxol-Induced Apoptosis. Mol. Cell.

[76] Bromberg J., Darnell J.E. (2000). The role of STATs in transcriptional control and their impact on cellular function. Oncogene.

[77] Santra S., Yang H., Stanley J.T., Holloway P.H., Moudgil B.M., Walter G., Mericle R.A. (2005). Rapid and effective labeling of brain tissue using TAT-conjugated CdSMn/ZnS quantum dots. Chem. Commun..

[78] Wadia J.S., Dowdy S.F. (2005). Transmembrane delivery of protein and peptide drugs by TAT-mediated transduction in the treatment of cancer. Adv. Drug Delivery Rev..

[79] Melnick A. (2007). Targeting aggressive B-cell lymphomas with cell-penetrating peptides. Biochem. Soc. Trans..

[80] Essler M., Ruoslahti E. (2002). Molecular specialization of breast vasculature: A breast-homing phage-displayed peptide binds to Aminopeptidase P in breast vasculature. Proc. Natl. Acad. Sci. USA.

[81] Elmquist A., Lindgren M., Bartfai T., Langel Ü. (2001). VE-Cadherin-derived cell-penetrating peptide, pVEC, with carrier functions. Exp. Cell Res..

[82] Myrberg H., Zhang L., Mäe M., Langel Ü. (2007). Design of a tumor-homing cell-penetrating peptide. Bioconjugate Chem..

[83] Menard S., Pupa S.M., Campiglio M., Tagliabue E. (2003). Biologic and therapeutic role of HER2 in cancer. Oncogene.

[84] Dayanidhi R., Paige J.B., Yee Mon T., Ann R. (2007). Role of chemokines in tumor growth. Cancer Lett..

[85] Daniel J.C., Chang H.K., Eugene C.B. (2003). Chemokines in the systemic organization of immunity. Immunol. Rev..

[86] Balkwill F. (2004). Cancer and the chemokine network. Nat. Rev. Cancer..

[87] Snyder E.L., Saenz C.C., Denicourt C., Meade B.R., Cui X.-S., Kaplan I.M., Dowdy S.F. (2005). Enhanced targeting and killing of tumor cells expressing the CXC chemokine receptor 4 by transducible anticancer peptides. Cancer Res..

[88] Zhou N., Luo Z., Luo J., Fan X., Cayabyab M., Hiraoka M., Liu D., Han X., Pesavento J., Dong C.-Z., Wang Y., An J., Kaji H., Sodroski J.G., Huang Z. (2002). Exploring the stereochemistry of CXCR4-peptide recognition and inhibiting HIV-1 entry with d-peptides derived from chemokines. J. Biol. Chem..

[89] Snyder E.L., Meade B.R., Saenz C.C., Dowdy S.F. (2004). Treatment of terminal Peritoneal Carcinomatosis by a transducible p53-activating peptide. PLoS Biol..

[90] Chen Y.-N.P., Sharma S.K., Ramsey T.M., Jiang L., Martin M.S., Baker K., Adams P.D., Bair K.W., Kaelin W.G. (1999). Selective killing of transformed cells by cyclin/cyclin-dependent kinase 2 antagonists. Proc. Natl. Acad. Sci. USA.

[91] Perea S.E., Reyes O., Puchades Y., Mendoza O., Vispo N.S., Torrens I., Santos A., Silva R., Acevedo B., Lopez E., Falcon V., Alonso D.F. (2004). Antitumor effect of a novel proapoptotic peptide that impairs the phosphorylation by the Protein Kinase 2 (Casein Kinase 2). Cancer Res..

[92] Yaylim I., Isbir T. (2002). Enhanced casein kinase II (CK II) activity in human lung tumours. Anticancer Res..

[93] Wang H., Davis A., Yu S., Ahmed K. (2001). Response of cancer cells to molecular interruption of the CK2 signal. Mol. Cell. Biochem..

[94] Ruzzene M., Penzo D., Pinna L.A. (2002). Protein kinase CK2 inhibitor 4,5,6,7-tetrabromobenzotriazole (TBB) induces apoptosis and caspase-dependent degradation of haematopoietic lineage cell-specific protein 1 (HS1) in Jurkat cells. Biochem. J..

[95] Mueller J., Gaertner F.C., Blechert B., Janssen K.-P., Essler M. (2009). Targeting of tumor blood vessels: A phage-displayed tumor-homing peptide specifically binds to matrix metalloproteinase-2-processed collagen IV and blocks angiogenesis *in vivo*. Mol. Cancer Res..

[96] Jiang T., Olson E.S., Nguyen Q.T., Roy M., Jennings P.A., Tsien R.Y. (2004). Tumor imaging by means of proteolytic activation of cell-penetrating peptides. Proc. Natl. Acad. Sci. USA.

[97] Roy R., Yang J., Moses M.A. (2009). Matrix metalloproteinases as novel biomarkers and potential therapeutic targets in human cancer. J. Clin. Oncol..

[98] Morris M.C., Deshayes S., Heitz F., Divita G. (2008). Cell-penetrating peptides: From molecular mechanisms to therapeutics. Biol. Cell..

[99] Friend S. (1994). p53: A glimpse at the puppet behind the shadow play. Science.

[100] Abarzua P., Losardo J.E., Gubler M.L., Spathis R., Lu Y.-A., Felix A., Nerri A. (1996). Restoration of the transcription activation function to mutant p53 in human cancer cells. Oncogene.

[101] Selivanova G., Iotsova V., Okan I., Fritsche M., Strom M., Groner B., Grafstrom R.C., Wiman K.G. (1997). Restoration of the growth suppression function of mutant p53 by a synthetic peptide derived from the p53 C-terminal domain. Nat. Med..

[102] Senatus P.B., Li Y., Mandigo C., Nichols G., Moise G., Mao Y., Brown M.D., Anderson R.C., Parsa A.T., Brandt-Rauf P.W., Bruce J.N., Fine R.L. (2006). Restoration of p53 function for selective Fas-mediated apoptosis in human and rat glioma cells *in vitro* and *in vivo* by a p53 COOH-terminal peptide. Mol. Cancer Ther..

[103] Li Y., Mao Y., Rosal R.V., Dinnen R.D., Williams A.C., Brandt-Rauf P.W., Fine R.L. (2005). Selective induction of apoptosis through the FADD/caspase-8 pathway by a p53 c-terminal peptide in human pre-malignant and malignant cells. Int. J. Cancer.

[104] Li Y., Rosal R.V., Brandt-Rauf P.W., Fine R.L. (2002). Correlation between hydrophobic properties and efficiency of carrier-mediated membrane transduction and apoptosis of a p53 C-terminal peptide. Biochem. Biophys. Res. Commun..

[105] Vocero-Akbani A.M., Heyden N.V., Lissy N.A., Ratner L., Dowdy S.F. (1999). Killing HIV-infected cells by transduction with an HIV protease-activated caspase-3 protein. Nat. Med..

[106] Semenza G.L. (2003). Targeting HIF-1 for cancer therapy. Nat. Rev. Cancer.

[107] Kizaka-Kondoh S., Tanaka S., Hiraoka M. (2009). Imaging and targeting of the hypoxia-inducible factor 1-active microenvironment. Toxicol. Pathol..

[108] Schwarze S.R., Ho A., Vocero-Akbani A., Dowdy S.F. (1999). *In vivo* Protein Transduction: Delivery of a Biologically Active Protein into the Mouse. Science.

[109] Hahn J.-S. (2009). The Hsp90 chaperone machinery: From structure to drug development. BMB Rep..

[110] Isaacs J.S., Xu W., Neckers L. (2003). Heat shock protein 90 as a molecular target for cancer therapeutics. Cancer Cell.

[111] Fortugno P., Beltrami E., Plescia J., Fontana J., Pradhan D., Marchisio P.C., Sessa W.C., Altieri D.C. (2003). Regulation of survivin function by Hsp90. Proc. Natl. Acad. Sci. USA.

[112] Plescia J., Salz W., Xia F., Pennati M., Zaffaroni N., Daidone M.G., Meli M., Dohi T., Fortugno P., Nefedova Y., Gabrilovich D.I., Colombo G., Altieri D.C. (2005). Rational design of shepherdin, a novel anticancer agent. Cancer Cell.

[113] Caughey B. (2003). Prion protein conversions: insight into mechanisms, TSE transmission barriers and strains. Br. Med. Bull..

[114] Lundberg P., Magzoub M., Lindberg M., Hällbrink M., Jarvet J., Eriksson L.E.G., Langel Ü., Gräslund A. (2002). Cell membrane translocation of the N-terminal (1-28) part of the prion protein. Biochem. Biophys. Res. Commun..

[115] Magzoub M., Oglęcka K., Pramanik A., Eriksson L.E.G., Gräslund A. (2005). Membrane perturbation effects of peptides derived from the N-termini of unprocessed prion proteins. Biochim. Biophys. Acta Biomembr..

[116] Lofgren K., Wahlstrom A., Lundberg P., Langel U., Graslund A., Bedecs K. (2008). Antiprion properties of prion protein-derived cell-penetrating peptides. FASEB J..

[117] Patel L., Zaro J., Shen W.-C. (2007). Cell-penetrating-peptides: Intracellular pathways and pharmaceutical perspectives. Pharm. Res..

[118] Heitz F., Morris M.C., Divita G. (2009). Twenty years of cell-penetrating peptides: From molecular mechanisms to therapeutics. Br. J. Pharmacol..

[119] Meade B.R., Dowdy S.F. (2008). Enhancing the cellular uptake of siRNA duplexes following noncovalent packaging with protein transduction domain peptides. Adv. Drug Delivery Rev..

[120] Moschos S.A., Jones S.W., Perry M.M., Williams A.E., Erjefalt J.S., Turner J.J., Barnes P.J., Sproat B.S., Gait M.J., Lindsay M.A. (2007). Lung selivery studies using siRNA conjugated to TAT(48-60) and Penetratin reveal peptide induced reduction in gene expression and induction of innate immunity. Bioconjug. Chem..

[121] Fonseca S.B., Pereira M.P., Kelley S.O. (2009). Recent advances in the use of cell-penetrating peptides for medical and biological applications. Adv. Drug Delivery Rev..

[122] Sebbage V. (2009). Cell-penetrating peptides and their therapeutic applications. Biosci. Horiz..

[123] Turner J.J., Jones S., Fabani M.M., Ivanova G., Arzumanov A.A., Gait M.J. (2007). RNA targeting with peptide conjugates of oligonucleotides, siRNA and PNA. Blood Cells Mol. Dis..

[124] Crombez L., Aldrian-Herrada G., Konate K., Nguyen Q.N., McMaster G.K., Brasseur R., Heitz F., Divita G. (2008). A new potent secondary amphipathic cell-penetrating peptide for siRNA delivery into mammalian cells. Mol. Ther..

[125] Eguchi A., Meade B.R., Chang Y.-C., Fredrickson C.T., Willert K., Puri N., Dowdy S.F. (2009). Efficient siRNA delivery into primary cells by a peptide transduction domain-dsRNA binding domain fusion protein. Nat. Biotech..

[126] El-Andaloussi S., Johansson H.J., Lundberg P., Langel Ü. (2006). Induction of splice correction by cell-penetrating peptide nucleic acids. J. Gen. Med..

[127] Hassane F.S., Ivanova G.D., Bolewska-Pedyczak E., Abes R., Arzumanov A.A., Gait M.J., Lebleu B., Garièpy J. (2009). A peptide-based dendrimer that enhances the splice-redirecting activity of PNA conjugates in cells. Bioconjugate Chem..

[128] Ivanova G.D., Arzumanov A., Abes R., Yin H., Wood M.J.A., Lebleu B., Gait M.J. (2008). Improved cell-penetrating peptide-PNA conjugates for splicing redirection in HeLa cells and exon skipping in mdx mouse muscle. Nucl. Acids Res..

[129] Moulton H.M., Fletcher S., Neuman B.W., McClorey G., Stein D.A., Abes S., Wilton S.D., Buchmeier M.J., Lebleu B., Iversen P.L. (2007). Cell-penetrating peptide-morpholino conjugates alter pre-mRNA splicing of DMD (Duchenne muscular dystrophy) and inhibit murine coronavirus replication *in vivo*. Biochem. Soc. Trans..

[130] Wust P., Hildebrandt B., Sreenivasa G., Rau B., Gellermann J., Riess H., Felix R., Schlag P.M. (2002). Hyperthermia in combined treatment of cancer. Lancet Oncol..

[131] Lebleu B., Moulton H.M., Abes R., Ivanova G.D., Abes S., Stein D.A., Iversen P.L., Arzumanov A.A., Gait M.J. (2008). Cell penetrating peptide conjugates of steric block oligonucleotides. Adv. Drug Deliv. Rev..

[132] Abes R., Moulton H.M., Clair P., Yang S.-T., Abes S., Melikov K., Prevot P., Youngblood D.S., Iversen P.L., Chernomordik L.V., Lebleu B. (2008). Delivery of steric block morpholino oligomers by (R-X-R)4 peptides: Structure-activity studies. Nucl. Acids Res..

[133] Sethuraman V.A., Na K., Bae Y.H. (2005). pH-responsive Sulfonamide/PEI system for tumor specific gene delivery: An *in vitro* study. Biomacromolecules.

[134] Sethuraman V.A., Bae Y.H. (2007). TAT peptide-based micelle system for potential active targeting of anti-cancer agents to acidic solid tumors. J. Control. Release.

[135] Tannock I.F., Rotin D. (1989). Acid pH in tumors and its potential for therapeutic exploitation. Cancer Res..

[136] Warburg O., Wind F., Negelein E. (1927). The metabolism of tumors in the body. J. Gen. Physiol..

[137] Semenza G.L., Artemov D., Bedi A., Bhujwalla Z., Chiles K., Feldser D., Laughner E., Ravi R., Simons J., Taghavi P., Zhong H., Goode J.A., Chadwick D.J. (2001). The metabolism of tumours: 70 years later. the Tumour Microenvironment: Causes and Consequences of Hypoxia and Acidity.

[138] Sawant R.M., Hurley J.P., Salmaso S., Kale A., Tolcheva E., Levchenko T.S., Torchilin V.P. (2006). "SMART" drug delivery systems: Double-targeted pH-responsive pharmaceutical nanocarriers. Bioconjugate Chem..

[139] Kale A.A., Torchilin V.P. (2007). Enhanced transfection of tumor cells *in vivo* using “Smart” pH-sensitive TAT-modified pegylated liposomes. J. Drug Targeting.

[140] Dewhirst M.W., Viglianti B.L., Lora-Michiels M., Hanson M., Hoopes P.J. (2003). Basic principles of thermal dosimetry and thermal thresholds for tissue damage from hyperthermia. Int. J. Hyperthermia.

[141] Meyer D.E., Shin B.C., Kong G.A., Dewhirst M.W., Chilkoti A. (2001). Drug targeting using thermally responsive polymers and local hyperthermia. J. Control. Release.

[142] Mart R.J., Osborne R.D., Stevens M.M., Ulijn R.V. (2006). Peptide-based stimuli-responsive biomaterials. Soft Matter.

[143] Hart D.S., Gehrke S.H. (2007). Thermally associating polypeptides designed for drug delivery produced by genetically engineered cells. J. Pharm. Sci..

[144] Meyer D.E., Kong G.A., Dewhirst M.W., Zalutsky M.R., Chilkoti A. (2001). Targeting a genetically engineered elastin-like polypeptide to solid tumors by local hyperthermia. Cancer Res..

[145] Bidwell G.L., Raucher D. (2005). Application of thermally responsive polypeptides directed against c-Myc transcriptional function for cancer therapy. Mol. Cancer Ther..

[146] Massodi I., Bidwell III G.L., Raucher D. (2005). Evaluation of cell penetrating peptides fused to elastin-like polypeptide for drug delivery. J. Control. Release.

[147] Manceur A.P., Driscoll B.D., Sun W., Audet J. (2008). Selective enhancement of the uptake and bioactivity of a TAT-conjugated peptide inhibitor of Glycogen Synthase Kinase-3. Mol. Ther..

[148] Rothbard J.B., Garlington S., Lin Q., Kirschberg T., Kreider E., McGrane P.L., Wender P.A., Khavari P.A. (2000). Conjugation of arginine oligomers to cyclosporin A facilitates topical delivery and inhibition of inflammation. Nat. Med..

[149] Koo J. (1998). A randomized, double-blind study comparing the efficacy, safety and optimal dose of two formulations of Cyclosporin, Neoral and Sandimmun, in patients with severe psoriasis. Br. J. Dermatol..

[150] Naeyaert J.M., Lachapelle J.M., Degreef H., de la Brassinne M., Heenen M., Lambert J. (1999). Cyclosporin in Atopic Dermatitis. Dermatology.

[151] McCusker C.T., Wang Y., Shan J., Kinyanjui M.W., Villeneuve A., Michael H., Fixman E.D. (2007). Inhibition of experimental allergic airways disease by local application of a cell-penetrating dominant-negative STAT-6 peptide. J. Immunol..

[152] Kuperman D.A., Huang X., Koth L.L., Chang G.H., Dolganov G.M., Zhu Z., Elias J.A., Sheppard D., Erle D.J. (2002). Direct effects of interleukin-13 on epithelial cells cause airway hyperreactivity and mucus overproduction in asthma. Nat. Med..

[153] Heckl S., Pipkorn R., Waldeck W., Spring H., Jenne J., von der Lieth C.-W., Corban-Wilhelm H., Debus J., Braun K. (2003). Intracellular visualization of prostate cancer using magnetic resonance imaging. Cancer Res..

[154] Louie A.Y., Huber M.M., Ahrens E.T., Rothbacher U., Moats R., Jacobs R.E., Fraser S.E., Meade T.J. (2000). *In vivo* visualization of gene expression using magnetic resonance imaging. Nat. Biotech..

[155] Lewin M., Carlesso N., Tung C.-H., Tang X.-W., Cory D., Scadden D.T., Weissleder R. (2000). Tat peptide-derivatized magnetic nanoparticles allow *in vivo* tracking and recovery of progenitor cells. Nat. Biotech..

[156] Sherr C.J. (1996). Cancer cell cycles. Science.

[157] Tan M.L., Choong P.F.M., Dass C.R. (2010). Recent developments in liposomes, microparticles and nanoparticles for protein and peptide drug delivery. Peptides.

[158] Malam Y., Loizidou M., Seifalian A.M. (2009). Liposomes and nanoparticles: nanosized vehicles for drug delivery in cancer. Trends Pharmacol. Sci..

[159] Fanciullino R., Ciccolini J. (2009). Liposome-encapsulated anticancer drugs: Still waiting for the magic bullet?. Curr. Med. Chem..

[160] Clark P.R., Hersh E.M. (1999). Cationic lipid-mediated gene transfer: current concepts. Curr. Opin. Mol. Ther..

[161] Ko Y.T., Hartner W.C., Kale A., Torchilin V.P. (2008). Gene delivery into ischemic myocardium by double-targeted lipoplexes with anti-myosin antibody and TAT peptide. Gene Ther..

[162] Sheikh F., Sontag D.P., Fandrich R.R., Kardami E., Cattini P.A. (2001). Overexpression of FGF-2 increases cardiac myocyte viability after injury in isolated mouse hearts. Am. J. Physiol. Heart Circ. Physiol..

[163] Jayakumar J., Suzuki K., Sammut I.A., Smolenski R.T., Khan M., Latif N., Abunasra H., Murtuza B., Amrani M., Yacoub M.H. (2001). Heat Shock Protein 70 gene transfection protects mitochondrial and ventricular function against ischemia-reperfusion injury. Circulation.

[164] Mack C.A., Patel S.R., Schwarz E.A., Zanzonico P., Hahn R.T., Ilercil A., Devereux R.B., Goldsmith S.J., Christian T.F., Sanborn T.A., Kovesdi I., Hackett N., Isom O.W., Crystal R.G., Rosengart T.K. (1998). Biologic bypass with the use of adenovirus-mediated gene transfer of the complementary deoxyribonucleic acid for vascular endothelial growth factor 121 improves myocardial perfusion and function in the ischemic porcine heart. J. Thorac. Cardiovasc. Surg..

[165] Keiji I., Yoshiki S., Satoru K.-S., Naomasa K., Nariaki M., Toshikazu N., Hikaru M. (2005). Gene transfection of hepatocyte growth factor attenuates the progression of cardiac remodeling in the hypertrophied heart. J. Thorac. Cardiovasc. Surg..

[166] Khaw B.A., Mattis J.A., Melincoff G., Strauss H.W., Gold H.K., Haber E. (1984). Monoclonal antibody to cardiac myosin: Imaging of experimental myocardial infarction. Hybridoma.

[167] Dass C.R., Choong P.F.M. (2009). Selective gene delivery for cancer therapy using cationic liposomes: *In vivo* proof of applicability. J. Control. Release.

[168] Leonie B., Klaas P., Grietje M., Dirk K.F.M. (1998). Targeting of sugar- and charge-modified albumins to fibrotic rat livers: The accessibility of hepatic cells after chronic bile duct ligation. J. Hepatol..

[169] Tuan Giam Chuang V., Kragh-Hansen U., Otagiri M. (2002). Pharmaceutical strategies utilizing recombinant human serum albumin. Pharm. Res..

[170] Zoltán S., Alisa E.K. (2004). Vascular endothelium and immune responses: Implications for inflammation and angiogenesis. Rheum. Dis. Clin. North Am..

[171] Temming K., Lacombe M., van der Hoeven P., Prakash J., Gonzalo T., Dijkers E.C.F., Orfi L., Keri G., Poelstra K., Molema G., Kok R.J. (2006). Delivery of the p38 MAPkinase inhibitor SB202190 to angiogenic endothelial cells: Development of novel RGD-Equipped and PEGylated drug-albumin conjugates using platinum(II)-based drug linker technology. Bioconjugate Chem..

[172] Temming K., Meyer D.L., Zabinski R., Senter P.D., Poelstra K., Molema G., Kok R.J. (2007). Improved efficacy of αvβ3-targeted albumin conjugates by conjugation of a novel Auristatin derivative. Mol. Pharmaceutics.

[173] El-Sayed A., Futaki S., Harashima H. (2009). Delivery of macromolecules using arginine-rich cell-penetrating peptides: Ways to overcome endosomal entrapment. AAPS J..

[174] Singh R., Kostarelos K. (2009). Designer adenoviruses for nanomedicine and nanodiagnostics. Trends Biotechnol..

[175] Zochowska M., Paca A., Schoehn G., Andrieu J.-P., Chroboczek J., Dublet B., Szolajska E. (2009). Adenovirus Dodecahedron, as a drug delivery vector. PLoS One.

[176] Ogris M., Wagner E. (2002). Targeting tumors with non-viral gene delivery systems. Drug Discov. Today.

[177] Järver P., Langel Ü. (2004). The use of cell-penetrating peptides as a tool for gene regulation. Drug Discov. Today.

[178] Mintzer M.A., Simanek E.E. (2008). Nonviral vectors for gene delivery. Chem. Rev..

[179] Torchilin V.P. (2005). Recent advances with liposomes as pharmaceutical carriers. Nat. Rev. Drug. Discov..

[180] Boeckle S., Wagner E. (2006). Optimizing targeted gene delivery: Chemical modification of viral vectors and synthesis of artificial virus vector systems. AAPS J..

[181] Kreppel F., Gackowski J., Schmidt E., Stefan K. (2005). Combined genetic and chemical capsid modifications enable flexible and efficient de- and retargeting of Adenovirus vectors. Mol. Ther..

[182] Frankel A.D., Pabo C.O. (1988). Cellular uptake of the tat protein from human immunodeficiency virus. Cell.

[183] Chen L., Harrison S.D. (2007). Cell-penetrating peptides in drug development: Enabling intracellular targets. Biochem. Soc. Trans..

[184] Moulton H.M., Moulton M.J., Kurreck J. (2008). Antisense morpholino oligomers and theirs peptide conjugates. Therapeutic Oligonucleotide.

[185] Juliano R.L., Alam R., Dixit V., Kang H.M. (2009). Cell-targeting and cell-penetrating peptides for delivery of therapeutic and imaging agents. Wiley Interdiscip. Rev. Nanomed. Nanobiotechnol..

